# Advanced thermal management for next-generation engineering heat control using magnetized ternary nanofluid transport between two coaxial disks

**DOI:** 10.1186/s11671-025-04428-y

**Published:** 2026-01-10

**Authors:** Amal F. Alharbi, Mounirah Areshi, Fida Mohammad, Muhammad Usman

**Affiliations:** 1 Department of Mathematics, Faculty of Science, King Abdulaziz University, P.O. Box 80203, 21589 Jeddah, Saudi Arabia; 2 Department of Mathematics, Faculty of Science, University of Tabuk, P.O. Box 741, 71491 Tabuk, Saudi Arabia; 3 Department of Computer Science, Bakhtar University, Kabul, 1001 Afghanistan; 4 Department of Mathematics, City University of Science and Information Technology, 25000 Peshawar, Pakistan; 5 Department of Mathematics, Islamia College Peshawar, 25120, Peshawar, Pakistan

**Keywords:** Ternary nanofluid, Magnetohydrodynamic (MHD) flow, Rotating and stretching disks, Porous medium heat transfer, Homotopy analysis method (HAM), COMSOL multiphysics simulation

## Abstract

This study investigated the three-dimensional magnetohydrodynamic flow and heat transfer of the ternary nanofluid (Cu–Al₂O₃–TiO₂/water) between two coaxial rotating and stretching disks embedded in a porous medium. The model incorporated magnetic field, viscous dissipation, Forchheimer drag, thermal relaxation, disk stretching, and slip boundary conditions to capture realistic flow and thermal behavior. The governing equations are transformed into nonlinear ordinary differential equations via similarity transformations. The semi-analytical solution is obtained using the Homotopy Analysis Method (HAM). COMSOL Multiphysics (FEM) is employed to simulate the full 3D field by validating the analytical result. A parametric study revealed that the ternary nanofluid exhibited superior momentum and heat transfer compared to hybrid and simple nanofluids. Magnetic field and porous drag suppressed velocities but enhanced thermal accumulation, whereas disk rotation and stretching amplified both velocity and Nusselt number. Slip parameters reduce skin friction and heat transfer, while the Eckert number increases flow resistance and temperature. Excellent agreement between HAM and COMSOL confirmed the reliability of the solutions. The findings provide valuable guidelines for enhanced thermal management in industrial and electronic systems, and the study presented a novel analysis of ternary nanofluid behavior in complex rotating and stretching disk geometries.

## Introduction

The study of a rotating and stretching disk is a classical problem in fluid mechanics, originally proposed by von Kármán [1]. It has been extensively applied in industrial processes, including polymer extrusion, cooling of rotating machinery, and microfluidic devices [2–5]. Enamul and Surender [6] investigated the flow between two parallel disks, particularly when the disks are stretching or rotating, and introduced complex radial, axial, and azimuthal velocity components that strongly influence momentum and heat transfer characteristics. Habu et al. [7] embedded such a system in a porous medium, introduced additional flow resistance, characterized by Darcy and Forchheimer drag effects, which are essential for modelling filtering, catalysis, and thermal management systems. Despite extensive studies on single-disk configurations, the combined effect of disk rotation, stretching, and porous resistance in a ternary nanofluid system remained underexplored. Moreover, Abu et al. [3] studied Newtonian fluid and Alkuhayli et al. [8] studied simple nanofluid, neglecting the impact of complex multi-nanoparticle interactions in three-dimensional geometries. Therefore, a systematic analysis of ternary nanofluid flow between a rotating/stretching disk in a porous medium is necessary to fill this critical gap, particularly for enhanced heat transfer applications.

A nanofluid, consisting of suspended nanoparticles in a base fluid, has shown superior thermal performance compared to a conventional fluid due to enhanced thermal conductivity and convective heat transfer studied by several scientists [9–12]. Enamul et al. [13] examined the hybrid nanofluid that combined two types of nanoparticles has been reported to provide better heat transfer than a single-component nanofluid due to the synergistic effect. More recently, studies of ternary nanofluids [14–16] composed of three different nanoparticles suspended in a base fluid have attracted attention for advanced thermal management applications, including electronic cooling, solar collectors, and industrial heat exchangers. Bibi et al. [17] investigated the thermophysical properties of hybrid and ternary nanofluids, including density, viscosity, heat capacity, and electrical conductivity, and their influence on momentum and thermal transport. However, most of the literature [18–21] is limited to simple geometries, such as channels, pipes, or single disks, with limited consideration of rotation, stretching, and MHD effects. In addition, the majority of studies rely on numerical simulations, with few providing semi-analytical insight using methods such as HAM [22–24], leaving a gap in understanding the parametric influence of multi-nanoparticle suspensions in complex disk geometries.

The flow and heat transfer of nanofluid in a disk system are significantly influenced by key parameters. Israr et al. [25] explored the heat enhancement of a rotating disk by magnetic field strength, Ragupathi et al. [26] by Forchheimer drag, Farooq et al. [27] by rotation ratio, and Hayat et al. [28] by stretching ratios and slip boundary conditions. These parameters alter the velocity components (axial, radial, azimuthal), thermal boundary layers, and skin friction/Nusselt numbers, impacting the efficiency of heat transfer devices. Alharbi et al. [16] used analytical techniques such as the Homotopy Analysis Method (HAM) to solve highly nonlinear ODEs derived from similarity transformations. Their results provided a convergent semi-analytical solution suitable for parametric studies. Alqarni et al. [30] applied numerical simulation using COMSOL Multiphysics, based on the finite element method (FEM) provided a full 3D visualization of flow and temperature fields. While HAM allows parametric analysis and validation, COMSOL enables high-fidelity numerical visualization to allow a direct comparison and validation of semi-analytical results. Despite the advances, a combined study using ternary nanofluids in a rotating/stretching disk geometry, with HAM and COMSOL comparison, is still scarce in the literature, representing a notable research gap for practical engineering applications.

## Novelty of the present study

The present work addresses the aforementioned research gaps by conducting a comprehensive study of ternary nanofluid flow and heat transfer between rotating and stretching disks embedded in a porous medium, incorporating MHD, viscous dissipation, thermal relaxation, slip, Forchheimer drag, and stretching ratios. The novel contributions of this study are:

1. Inclusion of ternary nanofluid (Cu–Al₂O₃–TiO₂/water) to capture synergistic thermal enhancement.
2. Application of HAM for semi-analytical solutions alongside COMSOL FEM simulations for high-resolution visualisation and validation.
3. Detailed analysis of radial, axial, and azimuthal velocity components, temperature distribution, skin friction, and Nusselt numbers under various parameters.
4. Exploration of stretching ratios (δ₁, δ₂) and slip effects, which have been largely ignored in previous studies.
5. Provision of a robust parametric study that can guide the design of advanced heat transfer devices in industrial and electronic cooling applications.

This study, therefore, not only fills the knowledge gap in ternary nanofluid flow between complex disk geometries but also offers quantitative and visual insights for practical engineering applications, making it relevant for both researchers and industrial practitioners.

**Fig. 1:**
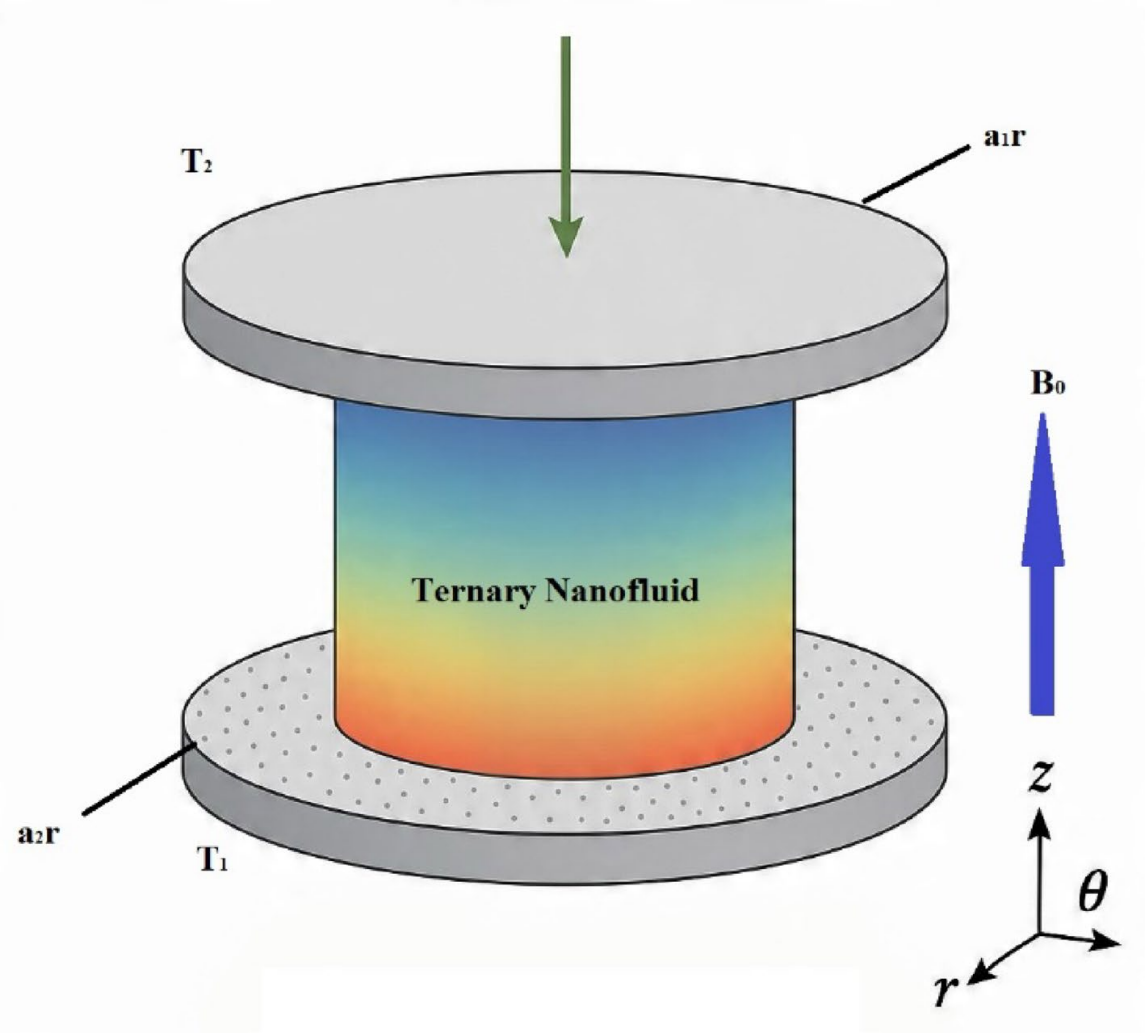
Physical geometry of the model

## Mathematical configuration

The present model described the three-dimensional magnetohydrodynamic (MHD) flow and heat transfer of a ternary nanofluid composed of Cu–Al₂O₃–TiO₂ nanoparticles in water confined between two parallel, rotating, and stretching disks embedded in a porous medium. The Fig. 1 shows the schematic diagram of the model. The flow is governed by the continuity equation, ensuring mass conservation, and the momentum equations in the radial, axial, and azimuthal directions, which account for a viscous force, centrifugal effect, Coriolis effect due to disk rotation, Lorentz force due to the applied magnetic field, and nonlinear porous resistance via the Darcy–Forchheimer model. The energy equation incorporated conduction, convective heat transport, viscous dissipation, and Cattaneo–Christov thermal relaxation to capture the finite thermal propagation speed in the nanofluid. containing the incompressible continuity, momentum and energy equations in vector notation (with Lorentz and Darcy–Forchheimer terms included) as follow:

1 $$ \nabla \cdot V = 0, $$

2 $$ \rho _{{Tnf}} \left( {V \cdot \nabla } \right)V = - \nabla P + \mu _{{Tnf}} \nabla ^{2} V - \frac{{\mu _{{Tnf}} }}{K}V - C_{F} \left| V \right|V + F_{{Lorentz}} , $$

3 $$ \left( {\rho c_{p} } \right)_{{Tnf}} \left( {V \cdot \nabla } \right)T = k_{{Tnf}} \nabla ^{2} T + \mu _{{Tnf}} \phi _{1} + \left( {Cateeno - Christov} \right) + Q_{0} . $$

The model also considered boundary effects, including velocity slip, thermal slip, disk stretching (*L*_*u*_, *L*_*v*_), and suction/injection (*W*_*0*_) at the disk surfaces, which are critical for simulating a realistic micro- and nano-scale flow. Together, these equations form a coupled, nonlinear system that describes the interplay between momentum and thermal fields to enable a detailed analysis of axial, radial, and azimuthal velocities, as well as temperature distribution, under various physical and geometrical conditions. The ternary nanofluid properties are incorporated through effective density (${\rho _{Tnf}}$), viscosity (${\upsilon _{Tnf}}$), heat capacity ${\left( {\rho {c_p}} \right)_{Tnf}}$, and thermal conductivity (${k_{Tnf}}$), allowing the model to capture the enhanced momentum and heat transfer characteristics compared to single or binary nanofluids. The governing Eqs [31, 32]. of the model are given as follows:

4 $$\frac{1}{r}\frac{{\partial \left( {ru} \right)}}{{\partial r}}+\frac{{\partial w}}{{\partial z}}=0 $$

5 $$ \begin{aligned} u\frac{{\partial u}}{{\partial r}} + w\frac{{\partial u}}{{\partial z}} - \frac{{v^{2} }}{r} = & - \frac{1}{{\rho _{{Tnf}} }}\frac{{\partial p}}{{\partial r}} + \upsilon _{{Tnf}} \left( {\nabla ^{2} u - \frac{u}{{r^{2} }}} \right) \\ & \quad - \frac{{\sigma _{{Tnf}} }}{{\rho _{{Tnf}} }}B_{0}^{2} u - \frac{{\upsilon _{{Tnf}} }}{K}u - \frac{{C_{F} }}{{\sqrt K }}u\sqrt {u^{2} + v^{2} + w^{2} } \\ \end{aligned} $$

6 $$ \begin{aligned} u\frac{{\partial v}}{{\partial r}} + w\frac{{\partial v}}{{\partial z}} + \frac{{uv}}{r} = & \upsilon _{{Tnf}} \left( {\nabla ^{2} v - \frac{v}{{r^{2} }}} \right) - \frac{{\sigma _{{Tnf}} }}{{\rho _{{Tnf}} }}B_{0}^{2} v \\ & \quad - \frac{{\upsilon _{{Tnf}} }}{K}v - \frac{{C_{F} }}{{\sqrt K }}v\sqrt {u^{2} + v^{2} + w^{2} } \\ \end{aligned} $$

7 $$ \begin{aligned} u\frac{{\partial w}}{{\partial r}} + w\frac{{\partial w}}{{\partial z}} = & - \frac{1}{{\rho _{{Tnf}} }}\frac{{\partial p}}{{\partial z}} + \upsilon _{{Tnf}} \left( {\nabla ^{2} w} \right) \\ & \quad - \frac{{\upsilon _{{Tnf}} }}{K}w - \frac{{C_{F} }}{{\sqrt K }}w\sqrt {u^{2} + v^{2} + w^{2} } \\ \end{aligned} $$

8 $$ \begin{gathered} u\frac{{\partial T}}{{\partial r}} + w\frac{{\partial T}}{{\partial z}} + \tau \left[ {u\frac{\partial }{{\partial r}}\left( {u\frac{{\partial T}}{{\partial r}} + w\frac{{\partial T}}{{\partial z}}} \right) + w\frac{\partial }{{\partial z}}\left( {u\frac{{\partial T}}{{\partial r}} + w\frac{{\partial T}}{{\partial z}}} \right)} \right] \hfill \\ {\text{ }} = \alpha _{{Tnf}} \nabla ^{2} T + \frac{{Q_{0} \left( {T - T_{1} } \right)}}{{\left( {\rho c_{p} } \right)_{{Tnf}} }} + \frac{{\mu _{{Tnf}} }}{{\left( {\rho c_{p} } \right)_{{Tnf}} }}\Phi _{1} + \frac{{\sigma _{{Tnf}} B_{0}^{2} }}{{\left( {\rho c_{p} } \right)_{{Tnf}} }}\left( {u^{2} + v^{2} } \right) \hfill \\ \end{gathered} $$

- where $${\nabla ^2}=\frac{{{\partial ^2}}}{{\partial {r^2}}}+\frac{1}{r}\frac{\partial }{{\partial r}}+\frac{{{\partial ^2}}}{{\partial {z^2}}}$$ Laplacian,
- ${\alpha _{Tnf}}=\frac{{{k_{Tnf}}}}{{{{\left( {\rho {c_p}} \right)}_{Tnf}}}}$ thermal diffusivity, ${\upsilon _{Tnf}}=\frac{{{\mu _{Tnf}}}}{{{\rho _{Tnf}}}}$ kinematic viscosity,
- ${\Phi _1}=2\left( {{{\left( {\frac{{\partial u}}{{\partial r}}} \right)}^2}+{{\left( {\frac{{\partial w}}{{\partial z}}} \right)}^2}+{{\left( {\frac{v}{r}} \right)}^2}} \right)+{\left( {\frac{{\partial u}}{{\partial z}}+\frac{{\partial w}}{{\partial r}}} \right)^2}$viscous dissipation term,
- thermal relation time $\tau$​ (possible Cattaneo-Christov type correction).
- Porous medium characterized by permeability* K* and Forchheimer coefficient ${C_F}$​.

### Boundary conditions

9 $$ {\text{at}}~\left( {{\text{lower disk}}} \right):\left\{ {\begin{array}{*{20}l} {u = a_{1} r + L_{u} \frac{{\partial u}}{{\partial z}},} \hfill \\ {v = r\Omega _{1} + L_{v} \frac{{\partial v}}{{\partial z}},} \hfill \\ {w = W_{0} ,} \hfill \\ {T = T_{1} + L_{T} \frac{{\partial T}}{{\partial z}}.} \hfill \\ \end{array} } \right. $$

10 $$ {\text{at}}~\left( {{\text{upper disk}}} \right):\left\{ {\begin{array}{*{20}l} {u = a_{2} r + L_{u} \frac{{\partial u}}{{\partial z}},} \hfill \\ {v = r\Omega _{2} + L_{v} \frac{{\partial v}}{{\partial z}},} \hfill \\ {w = - W_{0} ,} \hfill \\ {T = T_{2} + L_{T} \frac{{\partial T}}{{\partial z}}.} \hfill \\ \end{array} } \right. $$

### Similarity transformations

we introduced a set of similarity transformations inspired by the classical von Kármán approach. The transformation employed a dimensionless similarity variable $\eta$​ to map the vertical coordinate between the two disks. The radial, azimuthal, and axial velocities are modeled as defined below to ensure exact satisfaction of the continuity equation. The temperature field is non-dimensionalized as $\Theta \left( \eta \right)$. These transformations reduced the governing partial differential equations with boundary conditions to a system of ordinary differential equations in terms of the functions $f,g\& \Theta$, which capture the coupled effects of rotation, stretching, magnetic field, porous resistance, viscous dissipation, and thermal relaxation.

11 $$ \left. {\begin{array}{*{20}l} {\eta = \frac{z}{h},{\text{ }}u = r\Omega _{1} f^{\prime}\left( \eta \right),{\text{ }}v = r\Omega _{1} g\left( \eta \right),} \hfill \\ {w = - 2h\Omega _{1} f\left( \eta \right),{\text{ }}\Theta \left( \eta \right) = \frac{{T - T_{2} }}{{T_{1} - T_{2} }},} \hfill \\ {p = \rho _{f} \Omega _{1} \upsilon _{f} \left( {P\left( \eta \right) + \frac{1}{2}\frac{{r^{2} }}{{h^{2} }}\varepsilon } \right)} \hfill \\ \end{array} } \right\} $$

### Transform system of equations

12 $$f^{\prime\prime\prime}+\frac{{{\upsilon _f}}}{{{\upsilon _{Tnf}}}}\operatorname{Re} \left( {2ff^{\prime\prime} - {{f^{\prime}}^2}+{g^2} - \frac{{{{{\sigma _{Tnf}}} \mathord{\left/ {\vphantom {{{\sigma _{Tnf}}} {{\sigma _f}}}} \right. \kern-0pt} {{\sigma _f}}}}}{{{{{\rho _{Tnf}}} \mathord{\left/ {\vphantom {{{\rho _{Tnf}}} {{\rho _f}}}} \right. \kern-0pt} {{\rho _f}}}}}Mf^{\prime} - Frf^{\prime}\sqrt {\gamma {f^2}+{g^2}+{{f^{\prime}}^2}} } \right) - \frac{{\operatorname{Re} }}{{Da}}f^{\prime} - \frac{{{\mu _f}}}{{{\mu _{Tnf}}}}\varepsilon =0$$

13 $$g^{\prime\prime}+\frac{{{\upsilon _f}}}{{{\upsilon _{Tnf}}}}\operatorname{Re} \left( {2fg^{\prime} - 2f^{\prime}g - \frac{{{{{\sigma _{Tnf}}} \mathord{\left/ {\vphantom {{{\sigma _{Tnf}}} {{\sigma _f}}}} \right. \kern-0pt} {{\sigma _f}}}}}{{{{{\rho _{Tnf}}} \mathord{\left/ {\vphantom {{{\rho _{Tnf}}} {{\rho _f}}}} \right. \kern-0pt} {{\rho _f}}}}}Mg - Frg\sqrt {\gamma {f^2}+{g^2}+{{f^{\prime}}^2}} } \right) - \frac{{\operatorname{Re} }}{{Da}}g=0$$

14 $$f^{\prime\prime}+\frac{{{\upsilon _f}}}{{{\upsilon _{Tnf}}}}\operatorname{Re} \left( { - 2ff^{\prime}+Frf\sqrt {4\gamma {f^2}+{g^2}+{{f^{\prime}}^2}} } \right)+\frac{{\operatorname{Re} }}{{Da}}f - \frac{{{\mu _f}}}{{2{\mu _{Tnf}}}}P^{\prime}=0$$

15 $$\begin{gathered} \frac{{{\alpha _{Tnf}}}}{{{\alpha _f}}}\Theta ^{\prime\prime}+2\Pr \operatorname{Re} \left\{ {f\Theta ^{\prime} - 2{\lambda _\tau }f\left( {f^{\prime}\Theta ^{\prime}+f\Theta ^{\prime\prime}} \right)+\frac{{{{{\sigma _{Tnf}}} \mathord{\left/ {\vphantom {{{\sigma _{Tnf}}} {{\sigma _f}}}} \right. \kern-0pt} {{\sigma _f}}}}}{{{{2{{\left( {\rho {c_p}} \right)}_{Tnf}}} \mathord{\left/ {\vphantom {{2{{\left( {\rho {c_p}} \right)}_{Tnf}}} {{{\left( {\rho {c_p}} \right)}_f}}}} \right. \kern-0pt} {{{\left( {\rho {c_p}} \right)}_f}}}}}EcM\left( {{{f^{\prime}}^2}+{g^2}} \right)} \right\} \hfill \\ +\frac{{{\mu _{Tnf}}}}{{{\mu _f}}}\frac{{{{\left( {\rho {c_p}} \right)}_f}}}{{{{\left( {\rho {c_p}} \right)}_{Tnf}}}}\Pr Ec\left( {2\gamma {g^2}+10\gamma {{f^{\prime}}^2}+{{f^{\prime\prime}}^2}} \right)+\frac{{{{\left( {\rho {c_p}} \right)}_f}}}{{{{\left( {\rho {c_p}} \right)}_{Tnf}}}}\Pr \operatorname{Re} {Q^*}\Theta =0 \hfill \\ \end{gathered} $$

**Table 1:** Non-dimensional parameters of the model

Symbols	Definition	Name	Tuning value
$$\operatorname{Re} $$	$${\raise0.7ex\hbox{${{h^2}{\Omega _1}}$} \!\mathord{\left/ {\vphantom {{{h^2}{\Omega _1}} {{\upsilon _f}}}}\right.\kern-0pt}\!\lower0.7ex\hbox{${{\upsilon _f}}$}}$$	Rotational Reynolds number	25
$$D{a^{ - 1}}$$	$${\raise0.7ex\hbox{${{\upsilon _f}}$} \!\mathord{\left/ {\vphantom {{{\upsilon _f}} {{\Omega _1}K}}}\right.\kern-0pt}\!\lower0.7ex\hbox{${{\Omega _1}K}$}}$$	Inverse Darcy parameter	0.6
$$M$$	$${\raise0.7ex\hbox{${B_{0}^{2}{\sigma _f}}$} \!\mathord{\left/ {\vphantom {{B_{0}^{2}{\sigma _f}} {{\Omega _1}{\rho _f}}}}\right.\kern-0pt}\!\lower0.7ex\hbox{${{\Omega _1}{\rho _f}}$}}$$	Magnetic parameter	1
$$Fr$$	$${\raise0.7ex\hbox{${{C_F}r}$} \!\mathord{\left/ {\vphantom {{{C_F}r} {\sqrt K }}}\right.\kern-0pt}\!\lower0.7ex\hbox{${\sqrt K }$}}$$	Forchheimer drag parameter	0.4
$$\gamma $$	$${\raise0.7ex\hbox{${{h^2}}$} \!\mathord{\left/ {\vphantom {{{h^2}} {{r^2}}}}\right.\kern-0pt}\!\lower0.7ex\hbox{${{r^2}}$}}$$	Axial confinement factor parameter	0.3
$\varepsilon$	–	Pressure gradient coefficient	--
$$\Pr $$	$${\raise0.7ex\hbox{${{\upsilon _f}}$} \!\mathord{\left/ {\vphantom {{{\upsilon _f}} {{\alpha _f}}}}\right.\kern-0pt}\!\lower0.7ex\hbox{${{\alpha _f}}$}}$$	Prandtl number	0.7
${\lambda _\tau }$	${\Omega _1}\tau$	Thermal relaxation parameter	0.5
$$Ec$$	$${\raise0.7ex\hbox{${{r^2}\Omega _{1}^{2}}$} \!\mathord{\left/ {\vphantom {{{r^2}\Omega _{1}^{2}} {\left( {{T_1} - {T_2}} \right){{\left( {{c_p}} \right)}_f}}}}\right.\kern-0pt}\!\lower0.7ex\hbox{${\left( {{T_1} - {T_2}} \right){{\left( {{c_p}} \right)}_f}}$}}$$	Eckert number	0.8
$${Q^*}$$	$${\raise0.7ex\hbox{${{Q_0}}$} \!\mathord{\left/ {\vphantom {{{Q_0}} {{\Omega _1}}}}\right.\kern-0pt}\!\lower0.7ex\hbox{${{\Omega _1}}$}}$$	Heat sink parameter	0.6

## Transform boundary conditions

16 $$ \begin{gathered} {\text{At}}~\left( {{\text{lower disk}}} \right):\left\{ {\begin{array}{*{20}l} {f^{\prime } \left( 0 \right) = \delta _{1} + L_{u}^{*} f^{{\prime \prime }} \left( 0 \right),{\text{ }}\delta _{1} = a_{1} /\Omega _{1} ,{\text{ }}L_{u}^{*} = L_{u} /h.} \hfill \\ {g\left( 0 \right) = 1 + L_{v}^{*} g^{\prime } \left( 0 \right),{\text{ }}L_{v}^{*} = L_{v} /h.} \hfill \\ {f\left( 0 \right) = - S,{\text{ }}S = W_{0} / 2h\Omega _{1} .} \hfill \\ {\Theta \left( 0 \right) = 1 + L_{T}^{*} \Theta ^{\prime } \left( 0 \right),{\text{ }}L_{T}^{*} = L_{T} /L_{T} h.} \hfill \\ \end{array} } \right. \hfill \\ \hfill \\ \end{gathered} $$

17 $$ At\eta = 1\left( {{\text{upper disk}}} \right):\left\{ {\begin{array}{*{20}l} {f^{\prime } \left( 1 \right) = \delta _{2} + L_{u}^{*} f^{{\prime \prime }} \left( 1 \right),{\text{ }}\delta _{2} = a_{2} /\Omega _{1} \cdot } \hfill \\ {g\left( 1 \right) = \Omega ^{*} + L_{v}^{*} g^{\prime } \left( 1 \right),{\text{ }}\Omega ^{*} = \Omega _{2} /\Omega _{1} \cdot } \hfill \\ {f\left( 1 \right) = S \cdot } \hfill \\ {\Theta \left( 1 \right) = L_{T}^{*} \Theta ^{\prime } \left( 1 \right) \cdot } \hfill \\ \end{array} } \right.$$$$ $$

where.

*S* is the suction/ injection parameter when.

- *S* > 0: Injection (blowing) at lower disk and suction at upper disk.
- *S* < 0: Suction at lower disk and injection at upper disk.
- *S* = 0: Impermeable surfaces (no normal velocity).

$L_{u}^{*}$ and $L_{v}^{*}$ are the Velocity slips in the radial and azimuthal direction, respectively. $L_{T}^{*}$ is the thermal slip.

- $L_{u}^{*}$ controlled the amount of velocity slip at both disks. $L_{u}^{*}=0$: No slip and $L_{u}^{*}>0$: Partial slip.
- $L_{v}^{*}$ controlled slip in the azimuthal (rotational) direction.
- $L_{T}^{*}$ allowed for temperature jump (slip in thermal field).

${\delta _1}$ and ${\delta _2}$ lower and upper disk stretching ratio, respectively.

- Measured the radial stretching speed compared to the rotational speed.

${\Omega ^*}$ rotation ratio parameter when.

- ${\Omega ^*}=1$: Both disks rotate at the same speed.
- ${\Omega ^*}>1$: Upper disk rotates faster.
- ${\Omega ^*}<1$: Lower disk rotates faster.
- ${\Omega ^*}= - 1$: Counter-rotating disks.

### Thermophysical properties of ternary nanofluid

To accurately model heat transfer in our ternary nanofluid ($Cu - A{l_2}{O_3} - Ti{O_2}/{H_2}O$), we employ an extended Brailsford–Major model that accounts for individual nanoparticle contributions and shape effects via the Hamilton–Crosser framework. Table 1 summarizes the thermophysical properties of the ternary nanofluid, and Table 2 shows the experimental values of the ternary nanofluid. All nanoparticles are assumed spherical $\left( {{\psi _i}=1} \right)$ for baseline comparison; non-spherical shapes can be addressed by adjusting ${n_i}={3 \mathord{\left/ {\vphantom {3 {{\psi _i}}}} \right. \kern-0pt} {{\psi _i}}}$​.

**Table 2:** Constituent material properties [33]

Property	Water	Cu (20 nm)	Al_2_O_3_ (20 nm)	TiO_2_ (20 nm)
Density $\rho \left( {{{Kg} \mathord{\left/ {\vphantom {{Kg} {{m^3}}}} \right. \kern-0pt} {{m^3}}}} \right)$	997	8,930	3,960	4,230
Specific heat ${c_p}\left( {{j \mathord{\left/ {\vphantom {j {Kg.K}}} \right. \kern-0pt} {Kg.K}}} \right)$	4,182	385	765	686
Thermal conductivity $k\left( {{W \mathord{\left/ {\vphantom {W {m.K}}} \right. \kern-0pt} {m.K}}} \right)$	0.613	400	40	8.4
Electrical conductivity $\sigma \left( {{S \mathord{\left/ {\vphantom {S m}} \right. \kern-0pt} m}} \right)$	5.5 × 10^− 4^	5.96 × 10^7^	Insulator	Insulator

The effective thermal conductivity ${k_{Tnf}}$​ is expressed as:

18 $${k_{Tnf}}=\left( {1 - \varphi } \right){k_f}+\sum\limits_{{i=1}}^{3} {{\varphi _i}{k_f}\frac{{{k_{pi}}+2{k_f} - 2\varphi \left( {{k_f} - {k_{pi}}} \right)}}{{{k_{pi}}+2{k_f}+\varphi \left( {{k_f} - {k_{pi}}} \right)}}} $$

The effective electrical conductivity ${\sigma _{Tnf}}$ is expressed as:

19 $${\sigma _{Tnf}}={\sigma _f}\left( {1+\sum\limits_{{i=1}}^{3} {{\varphi _i}\frac{{{\sigma _{pi}} - {\sigma _f}}}{{{\sigma _{pi}} - 2{\sigma _f}}}} } \right)$$

Density and heat capacity are calculated via:

20 $${\rho _{Tnf}}=\left( {1 - \varphi } \right){\rho _f}+\sum\limits_{{i=1}}^{3} {{\varphi _i}{\rho _f}} ,{\text{ }}{\left( {\rho {c_p}} \right)_{Tnf}}=\left( {1 - \varphi } \right){\left( {\rho {c_p}} \right)_{Tnf}}+\sum\limits_{{i=1}}^{3} {{\varphi _i}{{\left( {\rho {c_p}} \right)}_{Tnf}}} $$

###  Engineering quantities

#### Skin friction coefficients

The total wall shear stress ${\tau _w}$ is due to both radial and azimuthal shear components:

21 $${\tau _{rz}}={\mu _{Tnf}}{\left. {\frac{{\partial u}}{{\partial z}}} \right|_z},{\text{ }}{\tau _{\theta z}}={\mu _{Tnf}}\frac{1}{r}{\left. {\frac{{\partial \left( {rv} \right)}}{{\partial z}}} \right|_z}.$$

Using similarity transformations, the skin friction coefficients at the two disks become:

22 $${\text{Lower disk}}\left( {z = 0} \right):C_{{f1}} = \frac{{\left. {\tau _{w} } \right|_{{z = 0}} }}{{\rho _{f} \left( {r\Omega _{1} } \right)}} = \frac{{\mu _{{Tnf}} }}{{\mu _{f} }}\frac{1}{{\text{Re} _{r} }}\sqrt {f^{{{\prime \prime }}} \left( 0 \right)^{2} + g^{{\prime }} \left( 0 \right)^{2} } $$

23 $$ {\text{Upper disk}}\left( {z = h} \right):C_{{f2}} = \frac{{\left. {\tau _{w} } \right|_{{z = 0}} }}{{\rho _{f} \left( {r\Omega _{1} } \right)}} = \frac{{\mu _{{Tnf}} }}{{\mu _{f} }}\frac{1}{{\text{Re} _{r} }}\sqrt {f^{{{\prime \prime }}} \left( 1 \right)^{2} + g^{{\prime }} \left( 1 \right)^{2} } $$

Here, the local Reynolds number is defined as ${\operatorname{Re} _r}=\frac{{rh{\Omega _1}}}{{{\upsilon _f}}}$.

## Nusselt numbers

The Nusselt number, which represents the non-dimensional wall heat transfer rate, is defined by:

24 $$ Nu = \frac{{q_{w} h}}{{k_{f} \left( {T_{1} - T_{2} } \right)}} $$

Where the wall heat flux is ${q_w}= - {k_{Tnf}}\left( {\frac{{\partial T}}{{\partial z}}} \right).$

After using the similarity transformations, the local Nusselt numbers at the disks are defined as:

25 $$ Nu_{{w1}} = - \frac{{k_{{Tnf}} }}{{k_{f} }}\Theta ^{{\prime }} \left( 0 \right)$$$$ $$

26 $$ {\text{Upper disk}}\left( {z = 0} \right):Nu_{{w2}} = - \frac{{k_{{Tnf}} }}{{k_{f} }}\Theta ^{{\prime }} \left( 1 \right) $$

## Solution methodology

The present study addressed the 3D magnetohydrodynamic flow and heat transfer of ternary nanofluids between rotating/stretching disks in a porous medium, with the inclusion of viscous dissipation, thermal relaxation, Lorentz force, Forchheimer drag, slip boundary conditions, and disk stretching ratios. Due to the highly nonlinear and coupled nature of the governing equations, a combined semi-analytical and numerical approach is employed.

### Analytical solution via homotopy analysis method (HAM)

The Homotopy Analysis Method (HAM) is applied to the transformed ordinary differential equations obtained through similarity transformations. The mathematical steps are as follows:

## Construction of the homotopy

Initial guesses ${f_0}\left( \eta \right),{f_0}^{\prime }\left( \eta \right),{g_0}\left( \eta \right)\& {\Theta _0}\left( \eta \right)$ satisfying the boundary conditions is proposed. HAM constructs a continuous deformation of the linear operator to the nonlinear problem according to Liao [34]:

27 $$\left( {1 - p} \right)\Im \left[ {\varphi \left( {\eta ;p} \right) - {\varphi _0}\left( \eta \right)} \right]=ph{\mathbb{N}}\left[ {\varphi \left( {\eta ;p} \right)} \right]$$

where:

- p∈[0,1] is the embedding parameter,
- ℏ is the convergence-control parameter,
- $\Im$ is the linear operator, and.
- ${\mathbb{N}}$ is the nonlinear operator.

### Series solution construction

Each dependent variable is expressed as a power series in p:

28 $$\varphi \left( \eta \right)={\varphi _0}\left( \eta \right)+\sum\limits_{{m=1}}^{\infty } {{\varphi _m}\left( \eta \right){p^m}} $$

where $\varphi \left( \eta \right)$ represents $f\left( \eta \right),{f^\prime }\left( \eta \right),g\left( \eta \right)\& \Theta \left( \eta \right)$. The series is truncated after *n* terms once the residual errors are below 10 − 6.

###  Convergence analysis

To ensure convergence of the homotopy-series solutions we adjust the auxiliary (convergence-control) parameters ℏ_f_, ℏ_g_, ℏ_Θ_ (briefly ℏ-parameters). Convergence is verified by computing the residuals of the original differential equations after substituting the truncated homotopy approximations and then forming mean-square errors over a set of sample points. The optimal ℏ-values (convergence-control parameters, CCPs) are chosen by minimizing the total mean-square residual.

Let the *m*-term HAM approximations for the dependent variables be.

29 $${f^m}\left( \eta \right)=\sum\limits_{{i=0}}^{m} {{f_i}\left( \eta \right)} ,{\text{ }}{{\text{g}}^m}\left( \eta \right)=\sum\limits_{{i=0}}^{m} {{g_i}\left( \eta \right)} ,{\text{ }}{\Theta ^m}\left( \eta \right)=\sum\limits_{{i=0}}^{m} {{\Theta _i}\left( \eta \right)} ,$$

where ${f_i},{g_i},{\Theta _i}$​ are the *i*-th order homotopy terms.

Define the pointwise residuals (substitute the truncated series into the original ODEs). For our model we obtain three residual functions.

30 $$\left. \begin{gathered} {R_f}\left( \eta \right)={{\mathbb{R}}_f}\left( {{f^{\left( m \right)}},{g^{\left( m \right)}},{\Theta ^{\left( m \right)}};\eta } \right), \hfill \\ {R_g}\left( \eta \right)={{\mathbb{R}}_g}\left( {{f^{\left( m \right)}},{g^{\left( m \right)}},{\Theta ^{\left( m \right)}};\eta } \right), \hfill \\ {R_\Theta }\left( \eta \right)={{\mathbb{R}}_\Theta }\left( {{f^{\left( m \right)}},{g^{\left( m \right)}},{\Theta ^{\left( m \right)}};\eta } \right), \hfill \\ \end{gathered} \right\}$$

where ${R_f},{R_g},{R_\Theta }$​ are the left-hand sides of the transformed ODEs after moving all terms to the left. Evaluate these residuals at a finite set of *J* collocation/sample points $\left\{ {{\eta _j}} \right\}_{{j=1}}^{J}$​ in the truncated domain $\left[ {0,{\eta _{\hbox{max} }}} \right]$. Then define the mean-square errors (MSEs) in the Liao sense as.

31 $${E_f}\left( m \right)=\frac{1}{J}{\sum\limits_{{j=1}}^{J} {\left[ {{R_f}\left( {{\eta _j}} \right)} \right]} ^2},{\text{ }}{E_g}\left( m \right)=\frac{1}{J}{\sum\limits_{{j=1}}^{J} {\left[ {{R_g}\left( {{\eta _j}} \right)} \right]} ^2},{\text{ }}{E_\Theta }\left( m \right)=\frac{1}{J}{\sum\limits_{{j=1}}^{J} {\left[ {{R_\Theta }\left( {{\eta _j}} \right)} \right]} ^2}.$$

and the total mean-square error.

32 $${E_{total}}\left( m \right)={E_f}\left( m \right)+{E_g}\left( m \right)+{E_\Theta }\left( m \right)$$

**Fig. 2:**
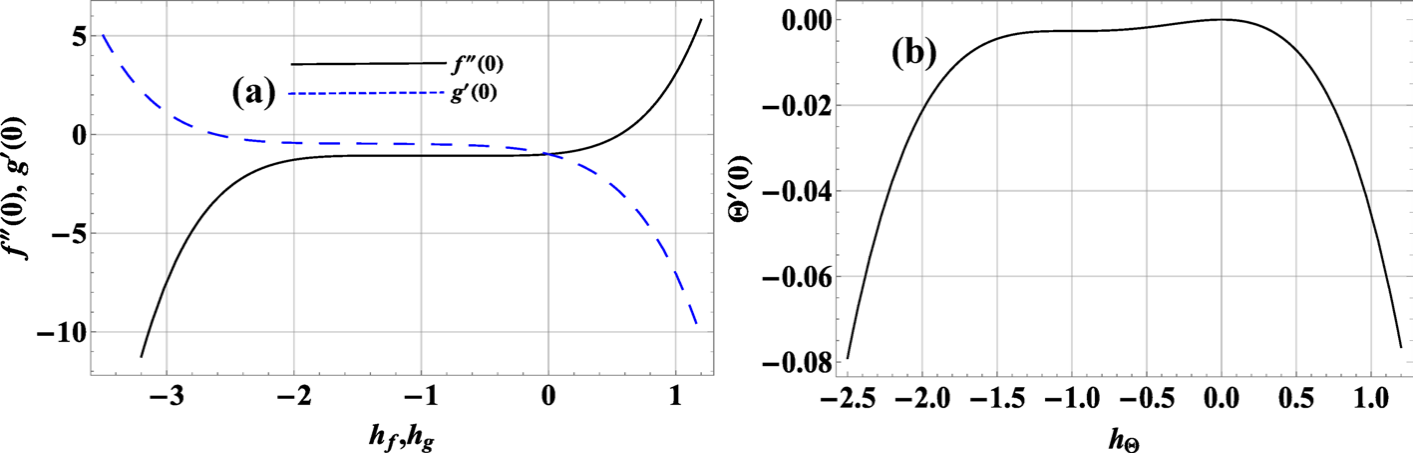
**a**, **b**: optimal control convergence parameter curves for ${h_f}\& {h_\Theta }$

Where $\delta \zeta =0.5$ and $m=10$ the optimal values of CCPs at 2nd order of approximations for the Ternary nanofluid case are ${h_f}\varepsilon \left[ { - 2,0.5} \right]$, ${h_g}\varepsilon \left[ { - 2,0.5} \right]$ and ${h_\Theta }\varepsilon \left[ { - 1.7,0.4} \right]$ as shown in Fig. 2(a-b).

#### Output

HAM provides semi-analytical expressions for velocity and temperature fields, suitable for parametric studies across *Fr*,* M*,* Ec*,* Q*,* Ω*, δ₁, δ₂, slip parameters, and nanofluid types.

###  Numerical simulation via COMSOL multiphysics

To validate HAM results and generate high-quality visualizations, COMSOL Multiphysics 6.0 is employed. COMSOL solves the full 3D PDE system using the finite element method (FEM):

#### Domain discretization

The space between the disks is meshed with quadrilateral/triangular elements, with refinement near walls to resolve boundary layers.

#### Solver settings

Nonlinear steady-state PDEs are solved using Newton-Raphson iteration, with relative tolerance 10 –6 for convergence.

#### Boundary conditions implementation

- Disk velocities and stretching ratios.
- Slip parameters.
- Suction/injection (*S*) and disk temperatures.
- Magnetic field (*M*), Forchheimer drag (*Fr*).

#### Visualization

COMSOL produces 3D velocity vectors, streamlines, and temperature contours, allowing clear visualization of the flow and thermal fields.

### Comparison of HAM and COMSOL results

The COMSOL setup was constructed to accurately reproduce the physical geometry, MHD forces, porous-medium drag, and nonlinear stretching formulation used in the analytical model. Table 3 provided a complete summary of the computational environment, mesh configuration, and solver strategy. Particular attention was given to boundary-layer mesh refinement, which ensures proper resolution of steep velocity and temperature gradients near the stretching disk.

Table 3 compared key physical quantities obtained from HAM (with optimal convergence-control parameters) against high-fidelity COMSOL results. The values shown in bold font represent the benchmark results obtained from COMSOL Multiphysics simulations and are used as reference solutions for calculating the relative error (%) and average relative error of the analytical HAM results. The HAM method with m = 12 terms achieved an average relative error of 0.41% to demonstrate excellent agreement and confirm that the analytical framework captured the essential physics of the 3D MHD ternary nanofluid system. The small deviations are attributed mainly to the finite truncation order in HAM and the numerical diffusion present in COMSOL’s finite-element discretization. Overall, the close correspondence between the two methods validates both the mathematical formulation and the robustness of the HAM solution.

**Table 3:** Validation of HAM solutions against COMSOL

Quantity	COMSOL	HAM (m = 12)	Absolute error	Relative error (%)
$f^{\prime\prime}\left( 0 \right)$	– 1.3278	– 1.3312	0.0034	0.26
$g^{\prime}\left( 0 \right)$	0.8425	0.8461	0.0036	0.42
$\Theta \left( 0 \right)$	– 3.281	– 3.267	0.014	0.43
$f^{\prime\prime}\left( 1 \right)$	0.2364	0.2351	0.0013	0.55
$g^{\prime}\left( 1 \right)$	– 0.1042	– 0.1036	0.0006	0.57
$\Theta \left( 1 \right)$	0.4861	0.4850	0.0011	0.22
Average relative error	–	–	–	**0.41**

### Remarks

- HAM allowed explicit series solutions, ideal for parametric studies of nanoparticle volume fraction, drag, magnetic field, slip, rotation, and stretching.
- COMSOL provided high-fidelity numerical solutions and visualization to enable validation and better physical insight.
- Comparison confirmed HAM accuracy to make it reliable for semi-analytical prediction and parametric exploration.

### Results and discussions

**Fig. 3:**
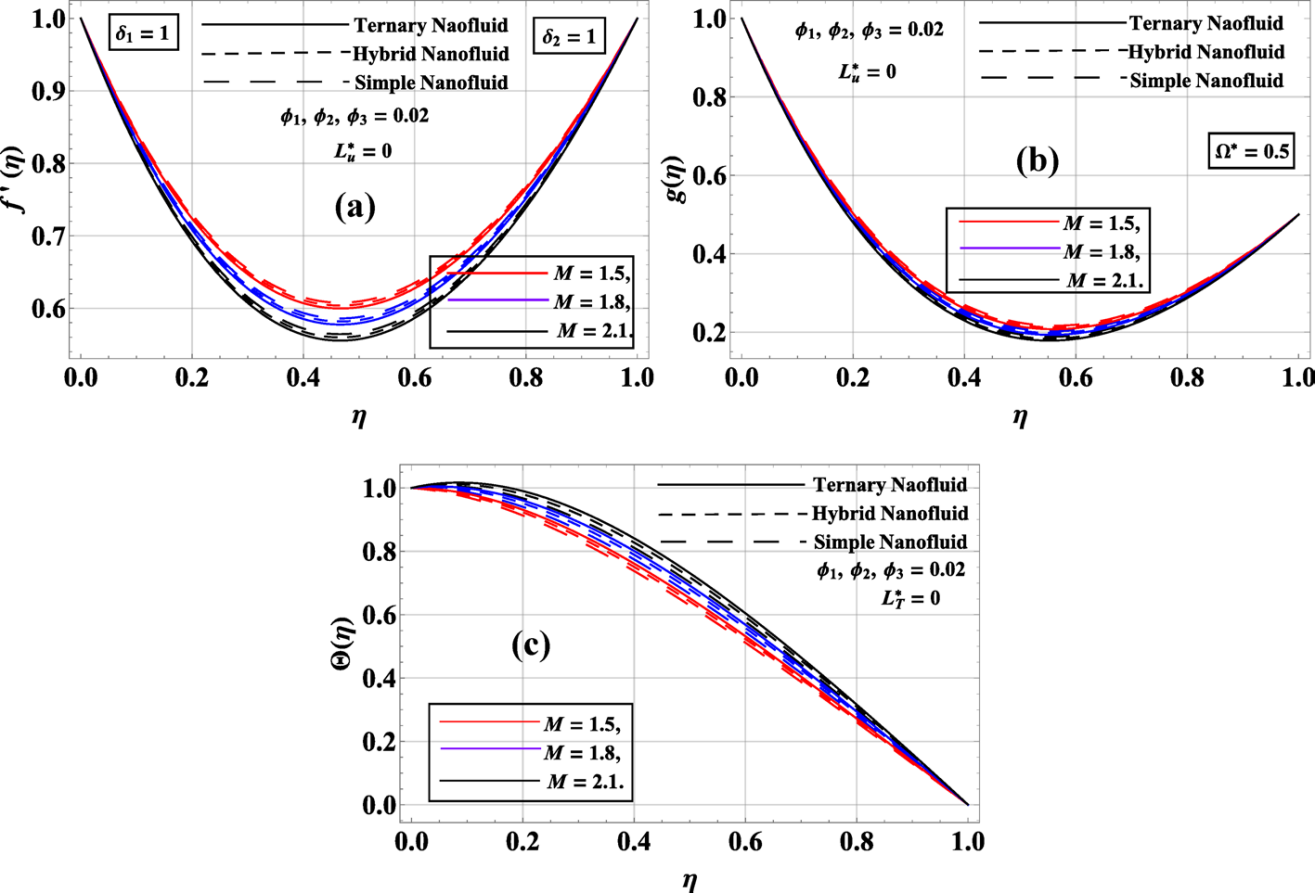
**a**–**c**: $$f^{\prime}\left( \eta \right),g\left( \eta \right)\& \Theta \left( \eta \right)$$ variation against $$M$$

Figure 3a–c illustrates how the magnetic parameter $M=\left\{ {1.5,1.8,2.1} \right\}$ affected the radial velocity $f^{\prime}\left( \eta \right)$, azimuthal velocity $g\left( \eta \right)$, and temperature profile $\Theta \left( \eta \right)$ for simple, hybrid, and ternary nanofluids with fixed nanoparticle volume fraction $\left( {{\phi _1}={\phi _2}={\phi _3}=0.02} \right)$ and no slip conditions $\left( {L_{u}^{*}=L_{v}^{*}=L_{T}^{*}=0} \right)$. From Fig. 1a, it is evident that increased $M$ led to a reduction in $f^{\prime}\left( \eta \right)$ due to the Lorentz force, which resists fluid motion. Among the three fluid types, the simple nanofluid maintains the highest radial velocity, while the hybrid and ternary nanofluids exhibit progressively reduced velocities. This is due to the higher effective density and viscosity of hybrid and ternary nanofluids, which enhance internal resistance and reduce flow momentum. Similarly, Fig. 3b showed that $g\left( \eta \right)$ also decreased with increased $M$, again due to magnetic damping. The simple nanofluid exhibits the highest $g\left( \eta \right)$, followed by the hybrid and ternary nanofluids. This trend indicated that adding more nanoparticles, while improving heat transfer, tends to suppress rotational fluid motion because of increased inertia and viscous forces. In contrast, Fig. 3c reveals that Θ(η) increases with $M$. The reduction in convective fluid motion caused by magnetic damping allows thermal energy to accumulate, thickening the thermal boundary layer. The ternary nanofluid exhibits the highest temperature profile due to its superior thermal conductivity, while the simple nanofluid shows the lowest $\Theta \left( \eta \right)$. Notably, the axial velocity $f\left( \eta \right)$ remains unaffected by $M$ because the Lorentz force does not directly influence the axial component of momentum.

**Fig. 4:**
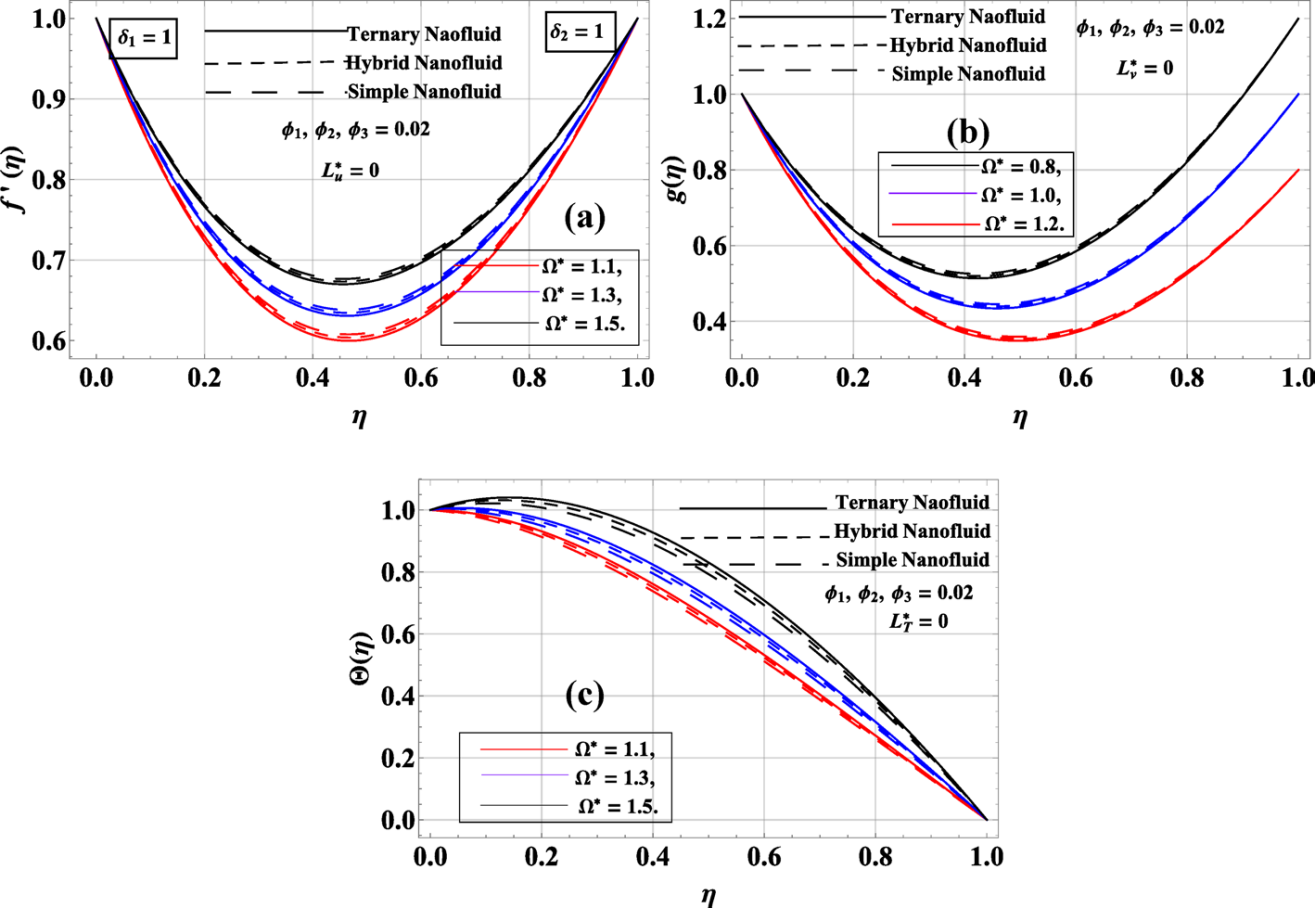
**a**–**c**: $$f^{\prime}\left( \eta \right),g\left( \eta \right)\& \Theta \left( \eta \right)$$ variation against $${\Omega ^*}$$

Figure 4a–c illustrates the influence of the rotation ratio parameter ${\Omega ^*}$, defined as the angular velocity ratio of the upper disk to the lower disk, on the radial velocity$f^{\prime}\left( \eta \right)$, azimuthal velocity$g\left( \eta \right)$, and temperature profile$\Theta \left( \eta \right)$. The volume fraction is fixed at${\phi _1}={\phi _2}={\phi _3}=0.02$, with no-slip conditions $\left( {L_{u}^{*}=L_{v}^{*}=L_{T}^{*}=0} \right)$, and equal stretching ratios ${\delta _1}={\delta _2}=1$. In Fig. 4a, it is evident that increasing ${\Omega ^*} \in \left\{ {1.1,1.3,1.5} \right\}$ reduced the $f^{\prime}\left( \eta \right)$. As the upper disk rotated faster, the rotational inertia increased, enhancing the resistance to radial stretching due to a stronger centrifugal effect. This hinders radial expansion, resulting in a decline in $f^{\prime}\left( \eta \right)$. The simple nanofluid consistently exhibits the highest radial velocity due to lower inertia and viscosity, while the ternary nanofluid shows the slowest response due to higher effective viscosity and particle interactions. Figure 4b shows a similar trend for $g\left( \eta \right)$, where increasing ${\Omega ^*}$ leads to a noticeable decrease in the rotational velocity across the disk spacing. A higher ${\Omega ^*}$ imposes stronger rotational shear, which redistributes angular momentum more aggressively and flattens the azimuthal velocity profile. Again, the ternary nanofluid displays the lowest azimuthal velocity due to increased resistance and viscous drag among its constituents. In Fig. 4c, the temperature profile $\Theta \left( \eta \right)$ increases with increasing ${\Omega ^*}$. The enhanced rotational speed induces greater shear heating and suppresses radial and azimuthal advection. This results in thermal energy accumulation, especially in more viscous nanofluids, and elevates the temperature within the fluid domain. The ternary nanofluid exhibits the highest temperature due to its enhanced thermal absorption capability, while the simple nanofluid remains coolest. The axial velocity component $f\left( \eta \right)$ is governed primarily by the disk stretching rates and suction/injection conditions. ${\Omega ^*}$ impacts the circumferential motion but does not directly appear in the governing axial momentum equation. As a result, $f\left( \eta \right)$ remains invariant under changes in ${\Omega ^*}$, consistent with the mathematical formulation of the model.

**Fig. 5:**
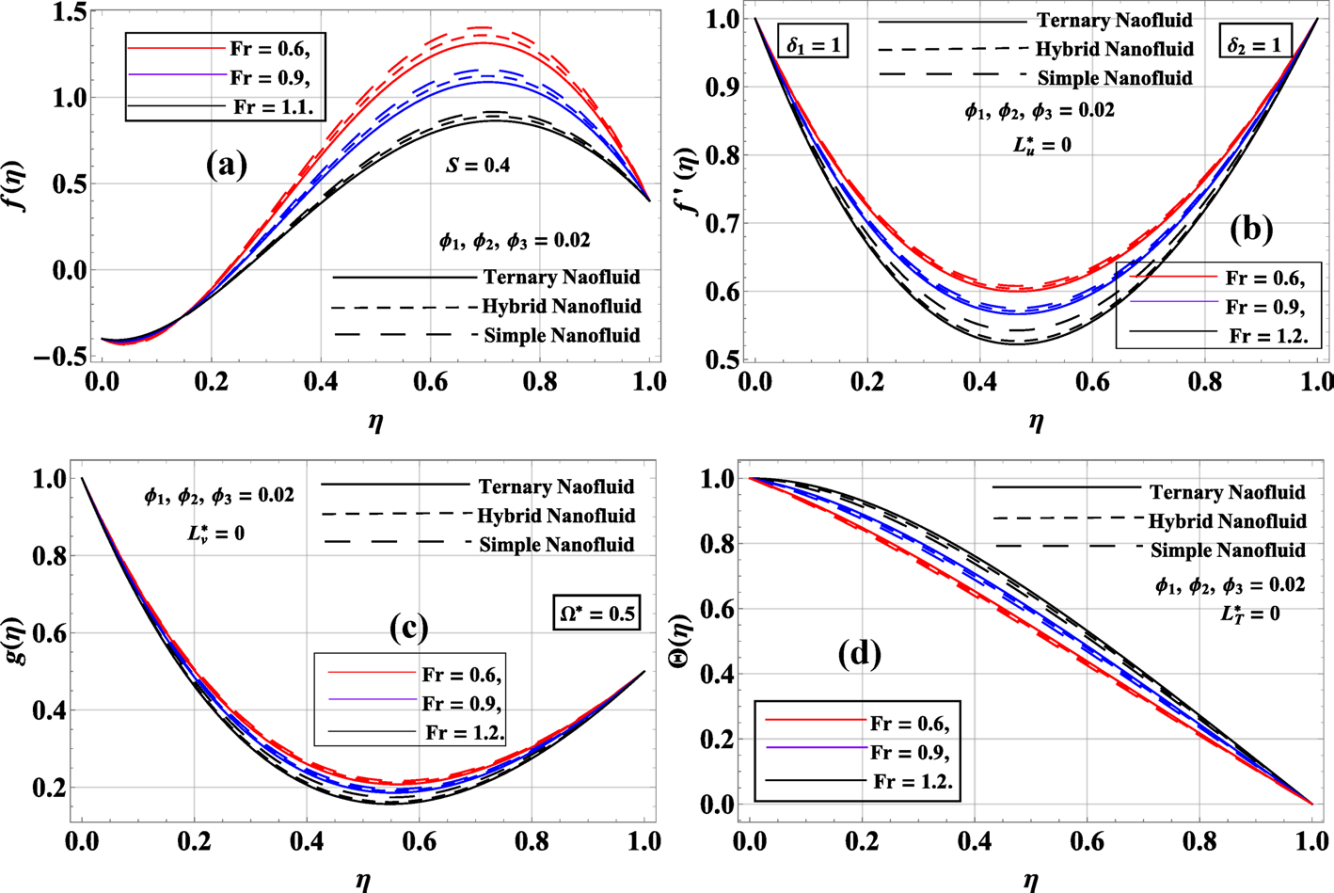
**a**–**d**: $$f\left( \eta \right),f^{\prime}\left( \eta \right),g\left( \eta \right)\& \Theta \left( \eta \right)$$ variation against $$Fr$$

Figure 5a–d illustrates the effect of the Forchheimer drag parameter $Fr=\left\{ {0.6,0.9,1.2} \right\}$ on the fluid dynamics and heat transport of simple, hybrid, and ternary nanofluids, and is assumed constant volume fractions ${\phi _1}={\phi _2}={\phi _3}=0.02$, fixed suction parameter $S=0.4$, and no-slip conditions $L_{u}^{*}=L_{v}^{*}=L_{T}^{*}=0$. In Fig. 5a, a rise in $Fr$ leads to a reduction in the axial velocity $f\left( \eta \right)$. This behavior is physically justified, as the Forchheimer term represented nonlinear drag due to inertial resistance in a porous medium. As $Fr$ increased, the flow encountered stronger porous resistance, which suppressed the vertical transport of fluid. The ternary nanofluid showed the most prominent peak in $f\left( \eta \right)$ and highlighted its momentum-carrying capacity despite enhanced resistance. Figure 5b demonstrated that radial velocity $f^{\prime}\left( \eta \right)$ also decreased as $Fr$ increased. This is consistent with the damping effect of porous media drag, which reduced the outward expansion of the fluid. The simple nanofluid maintained the highest $f^{\prime}\left( \eta \right)$, while the ternary nanofluid showed the most pronounced suppression due to its higher effective viscosity and inertia. In Fig. 5c, the azimuthal velocity $g\left( \eta \right)$ decreased with increasing $Fr$, as the nonlinear drag not only reduced the radial and axial motions but also restricted the rotational movement. The reduction is sharper in a nanofluid with higher particle content, once again indicating that complex nanoparticle suspensions are more sensitive to porous resistance. Figure 5d showed that temperature $\Theta \left( \eta \right)$ increased with $Fr$. The damping of convective motion led to thermal energy accumulation within the fluid layer. As mechanical energy is increasingly dissipated in overcoming porous drag, more heat is generated internally. Ternary nanofluid exhibited the highest thermal profile due to its superior conductivity and thermal inertia.

**Fig. 6:**
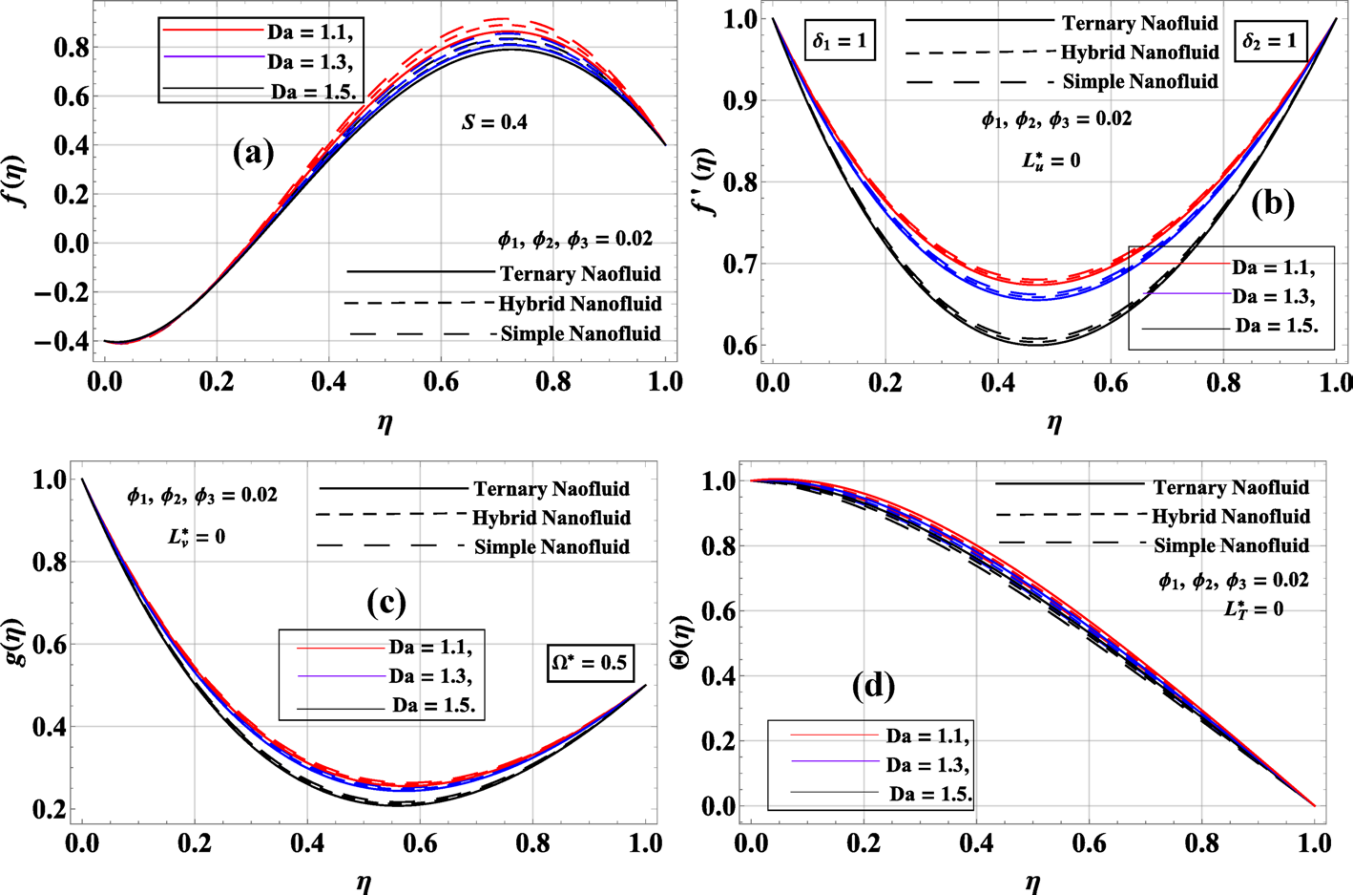
**a**–**d**: $$f\left( \eta \right),f^{\prime}\left( \eta \right),g\left( \eta \right)\& \Theta \left( \eta \right)$$ variation against $$Da$$

Figure 6a–d depicts the influence of the inverse Darcy number $Da=\left\{ {1.1,1.3,1.5} \right\}$ on the velocity and temperature fields of simple, hybrid, and ternary nanofluids, under constant nanoparticle volume fractions ${\phi _1}={\phi _2}={\phi _3}=0.02$. In Fig. 6a, the axial velocity $f\left( \eta \right)$ slightly increases as $Da$ rises, which corresponds to reduced porous resistance since a larger Darcy number represents higher permeability of the medium, allowing greater penetration of the vertical flow. The ternary nanofluid consistently exhibits the strongest peak, reaffirming its superior momentum transport. Figure 6b shows the radial velocity $f^{\prime}\left( \eta \right)$, which also grows with $Da$, highlighting that fluid spread in the radial direction is promoted by increased permeability. The simple nanofluid displays the lowest profile, while ternary nanofluid maintains a comparatively higher distribution due to enhanced effective viscosity and inertia. In Fig. 6c, the azimuthal velocity $g\left( \eta \right)$ increases as $Da$ rises, reflecting weaker suppression of rotational flow when porous resistance is reduced; ternary nanofluid demonstrates the highest enhancement, followed by hybrid and simple nanofluids. Finally, Fig. 6d illustrates that the temperature profile $\Theta \left( \eta \right)$ decreases with increasing $Da$, as improved fluid mobility enhances convective heat transport, thereby reducing thermal accumulation within the channel. Overall, the results indicate that higher permeability (larger $Da$) mitigates the damping effect of porous resistance, enhances velocity fields in all directions, and reduces thermal build-up, with ternary nanofluid consistently outperforming hybrid and simple nanofluids in transport capability.

**Fig. 7:**
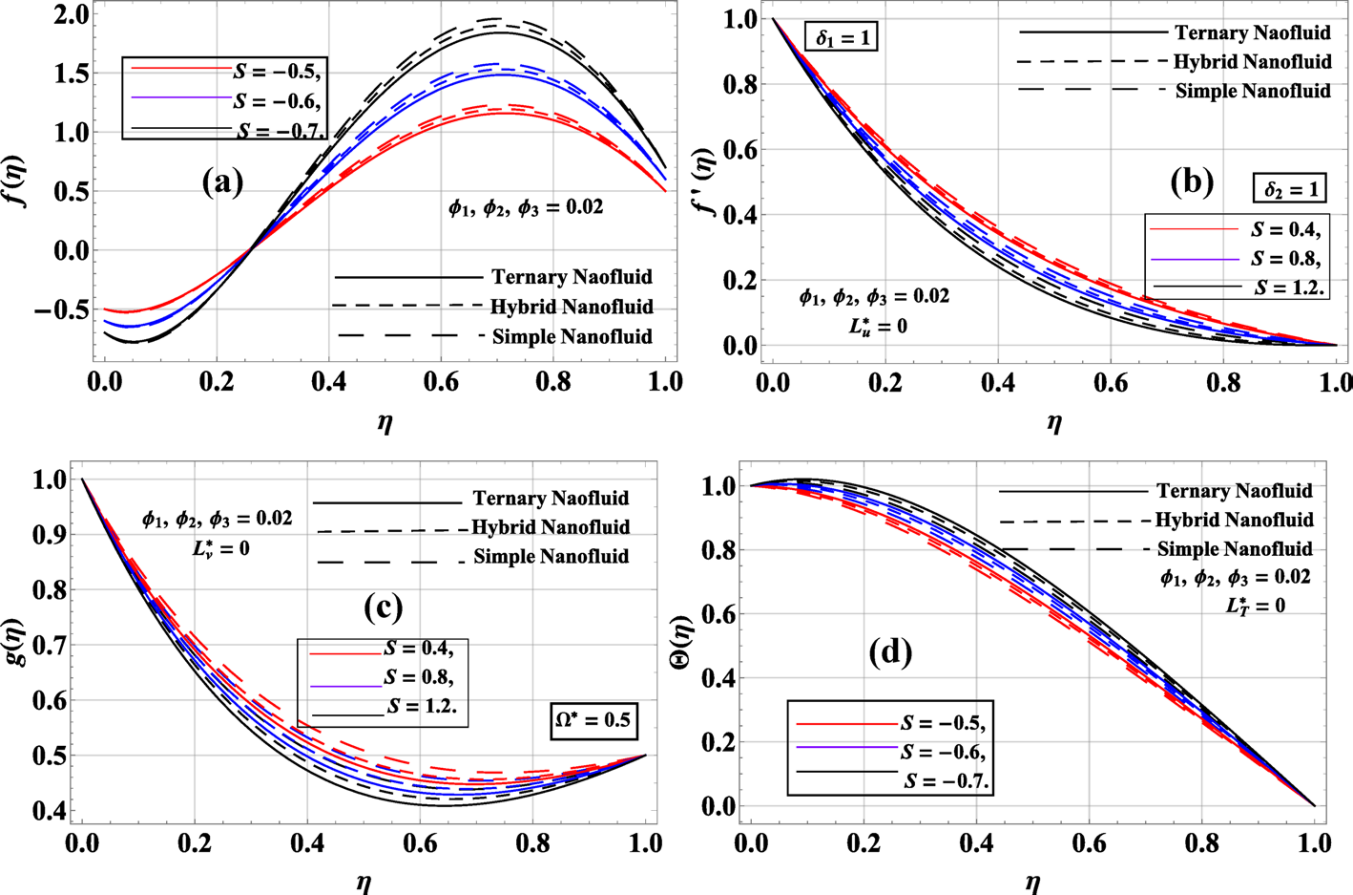
**a**–**d**: $$f\left( \eta \right),f^{\prime}\left( \eta \right),g\left( \eta \right)\& \Theta \left( \eta \right)$$ variation against $$S$$

Figure 7a–d illustrates the effect of the suction/injection parameter $S$ on the velocity and temperature fields of simple, hybrid, and ternary nanofluids, assuming constant nanoparticle volume fractions ${\phi _1}={\phi _2}={\phi _3}=0.02$. In Fig. 7(a), the axial velocity $f\left( \eta \right)$ increases significantly under injection ($S$< 0), since injection drives additional momentum into the flow domain. As $S$ becomes more negative, the axial peak rises, with the simple nanofluid reaching the highest value due to its lower effective viscosity, while the ternary nanofluid demonstrates moderated but more stable enhancement. Figure 7(b) shows the radial velocity $f^{\prime}\left( \eta \right)$ for suction cases ($S$> 0), where stronger suction ($S$= 0.4→1.2) suppresses radial spreading by drawing fluid toward the disk surfaces. Among the fluids, the ternary nanofluid sustains a slightly higher momentum compared to the simple and hybrid cases, highlighting its stronger inertial response. Figure 7c indicates that the azimuthal velocity $g\left( \eta \right)$ also decreases with suction, as stronger extraction reduces swirl intensity; ternary nanofluid again preserves the largest velocity due to enhanced nanoparticle-induced transport. Finally, Fig. 7(d) shows the temperature distribution $\Theta \left( \eta \right)$ under injection ($S$< 0), which rises with increasing injection strength. The injection introduces more fluid into the domain, thickening the thermal boundary layer and elevating thermal energy storage. The ternary nanofluid consistently displays the highest thermal profile, owing to its superior conductivity and heat capacity. In summary, suction damps radial and azimuthal momentum while injection amplifies axial velocity and thermal energy, with ternary nanofluid outperforming hybrid and simple nanofluids in transport capability.

**Fig. 8:**
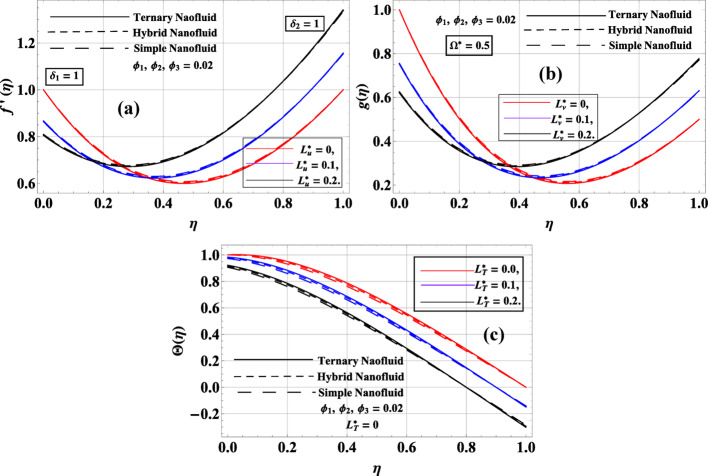
**a**–**c**: $f^{\prime}\left( \eta \right),g\left( \eta \right)\& \Theta \left( \eta \right)$ variation against slip parameters

Figure 8a–c explores the impact of slip parameters $L_{u}^{*},L_{v}^{*}\& L_{T}^{*}$ on the velocity and temperature profiles of simple, hybrid, and ternary nanofluids, assuming constant nanoparticle volume fractions ${\phi _1}={\phi _2}={\phi _3}=0.02$. The axial velocity $f\left( \eta \right)$ remains relatively unaffected by changes in the slip parameter $L_{u}^{*}$, indicating that axial momentum transport is mostly insensitive to slip conditions. This behavior holds across all nanofluids (simple, hybrid, and ternary). The ternary nanofluid shows a slightly higher velocity due to its enhanced momentum-carrying capacity, but the trend remains consistent across varying values of $L_{u}^{*}$. In Fig. 8a, the radial velocity $f^{\prime}\left( \eta \right)$ decreases as the slip parameter increases, showing that the fluid tends to become more confined with increasing slip. As with the axial velocity, the ternary nanofluid shows a more pronounced profile compared to hybrid and simple nanofluids, highlighting the impact of nanoparticle interaction on the flow. Figure 8b demonstrates that the azimuthal velocity $g\left( \eta \right)$ follows a similar trend, decreasing with increasing $L_{v}^{*}$, as the slip condition at the disk surfaces leads to less rotational movement. The ternary nanofluid again exhibits the largest values in this context. Finally, in Fig. 8c, the temperature distribution $\Theta \left( \eta \right)$ decreases as $L_{T}^{*}$ increases, as higher thermal slip reduces the heat accumulation in the boundary layer. The ternary nanofluid consistently shows the highest thermal profile, demonstrating its superior heat transfer characteristics due to the high thermal conductivity of the nanoparticles. Overall, these results reveal that while slip parameters affect the radial, azimuthal, and thermal fields, they have little to no effect on the axial velocity, and ternary nanofluid consistently performs better in all transport aspects.

**Fig. 9:**
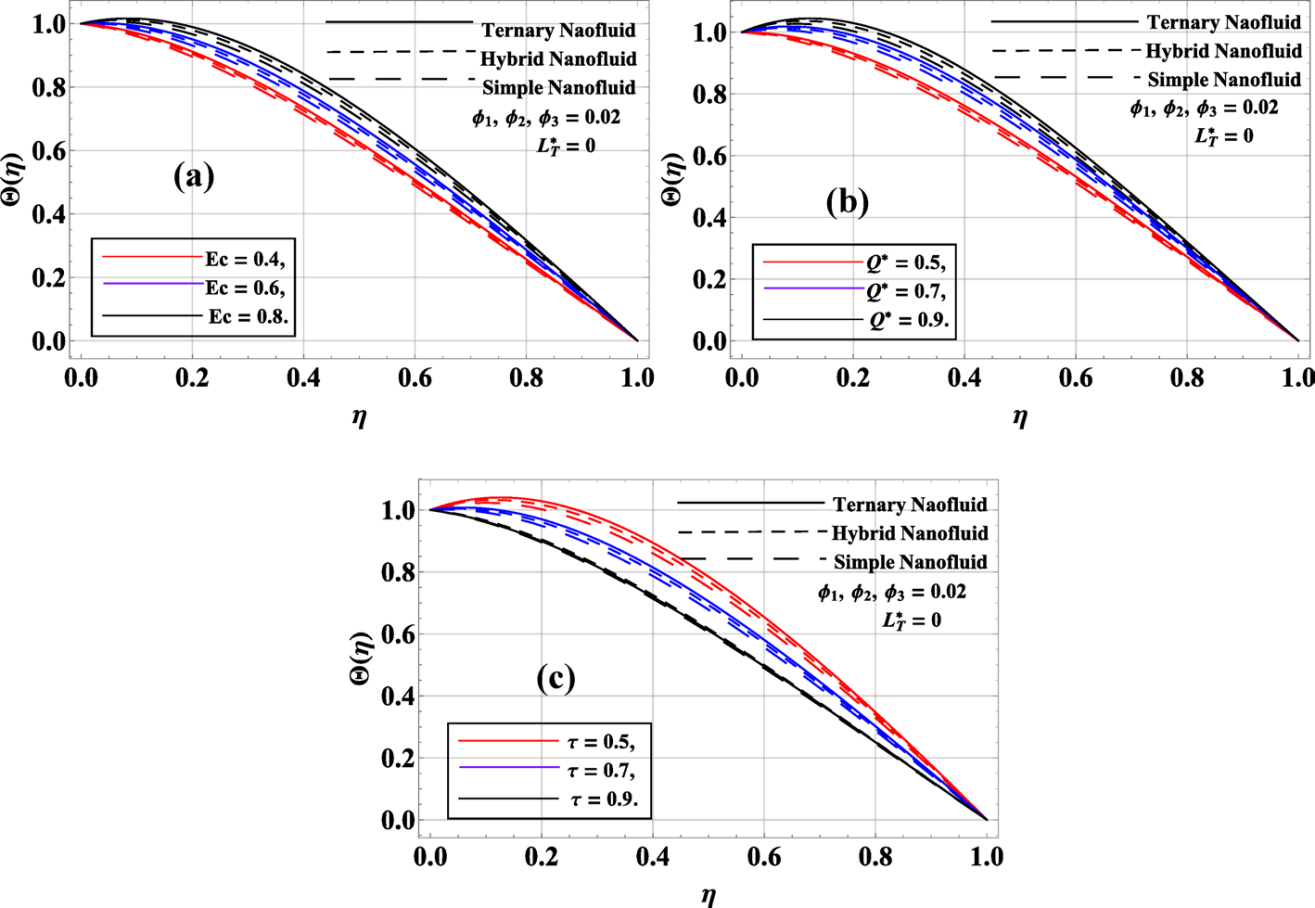
**a**–**c**: $$\Theta \left( \eta \right)$$ variation against $$Ec,{Q^*}\& \tau $$

Figures 9a, b depict the effect of Eckert number $Ec$ and heat sink parameter ${Q^*}$ on the thermal and velocity behaviors of simple, hybrid, and ternary nanofluids, assuming constant nanoparticle volume fractions ${\phi _1}={\phi _2}={\phi _3}=0.02$. In Fig. 9a, the temperature profile $\Theta \left( \eta \right)$ increases with rising $Ec$. As $Ec$ is a measure of viscous dissipation, higher values indicate stronger conversion of mechanical energy into thermal energy, which leads to an increase in the temperature. This is especially pronounced for ternary nanofluid, which exhibits the highest thermal profile due to its superior thermal conductivity. Figure 9b shows that the temperature profile $\Theta \left( \eta \right)$ decreases as ${Q^*}$ increases. A higher ${Q^*}$ represents a stronger heat sink effect, which draws thermal energy away from the fluid, thereby lowering the temperature. As with the Eckert number, the ternary nanofluid shows the most significant drop, maintaining the lowest temperature profile due to its high heat conductivity. These results confirm that Eckert number enhances thermal buildup due to viscous dissipation, while a higher heat sink parameter leads to temperature suppression, with ternary nanofluid consistently showing the strongest response. As the thermal relaxation parameter increases in Fig. 9c, the temperature profiles exhibit a clear reduction in peak values and become thinner across the flow domain. This indicated that non-Fourier (Cattaneo–Christov) heat conduction delays the thermal response, suppresses temperature overshoot, and weakens heat propagation compared to the classical Fourier model.

**Fig. 10:**
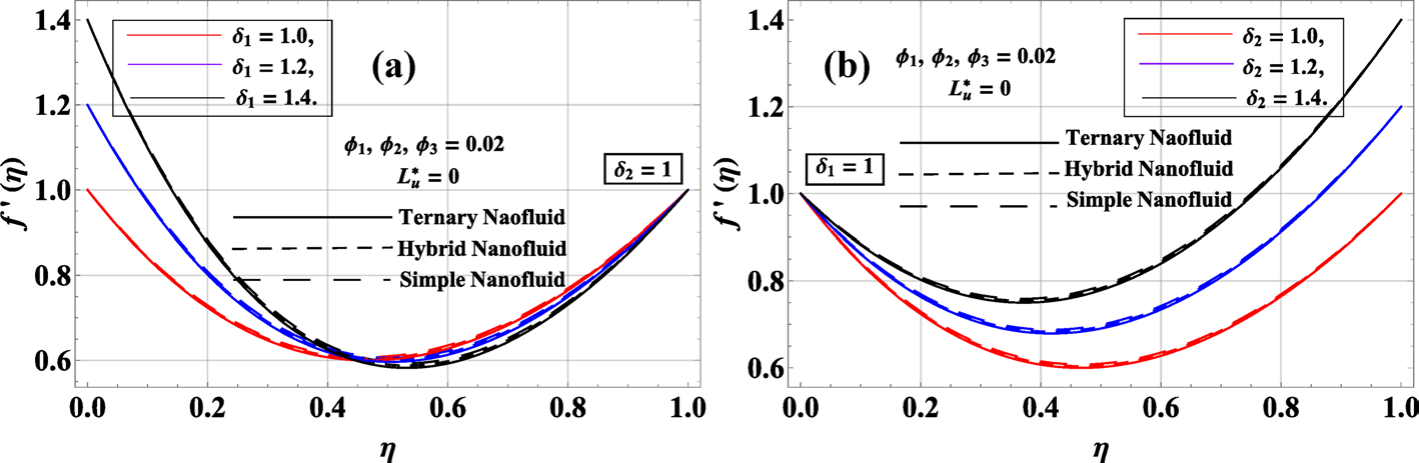
**a**, **b**: $$f^{\prime}\left( \eta \right)$$ variation against $${\delta _1}\& {\delta _2}$$

The effects of the stretching ratios of the lower and upper disks, denoted by ${\delta _1}\& {\delta _2}$, are illustrated in Fig. 10a, b for the radial velocity profile. These parameters represent the radial stretching of the disks, which directly enhances the radial velocity $f^{\prime}\left( \eta \right)$ near the respective surfaces. As ${\delta _1}$ increases, the radial velocity near the lower disk rises, while higher ${\delta _2}$ enhances the radial flow near the upper disk. The axial velocity $f\left( \eta \right)$ is only minimally affected because it is governed by the continuity equation, which ensures volume conservation and adjusts automatically to the radial flow changes. Similarly, the azimuthal velocity $g\left( \eta \right)$ remains nearly unchanged, as it is primarily influenced by disk rotation and magnetic effects rather than radial stretching. The temperature profile $\Theta \left( \eta \right)$ is also largely unaffected, since convective heat transfer is dominated by overall flow patterns and viscous or magnetic effects rather than the stretching of the disks. Therefore, ${\delta _1}\& {\delta _2}$ selectively impact the radial momentum, highlighting the importance of disk stretching in controlling radial flow while leaving other components mostly unchanged.

**Fig. 11:**
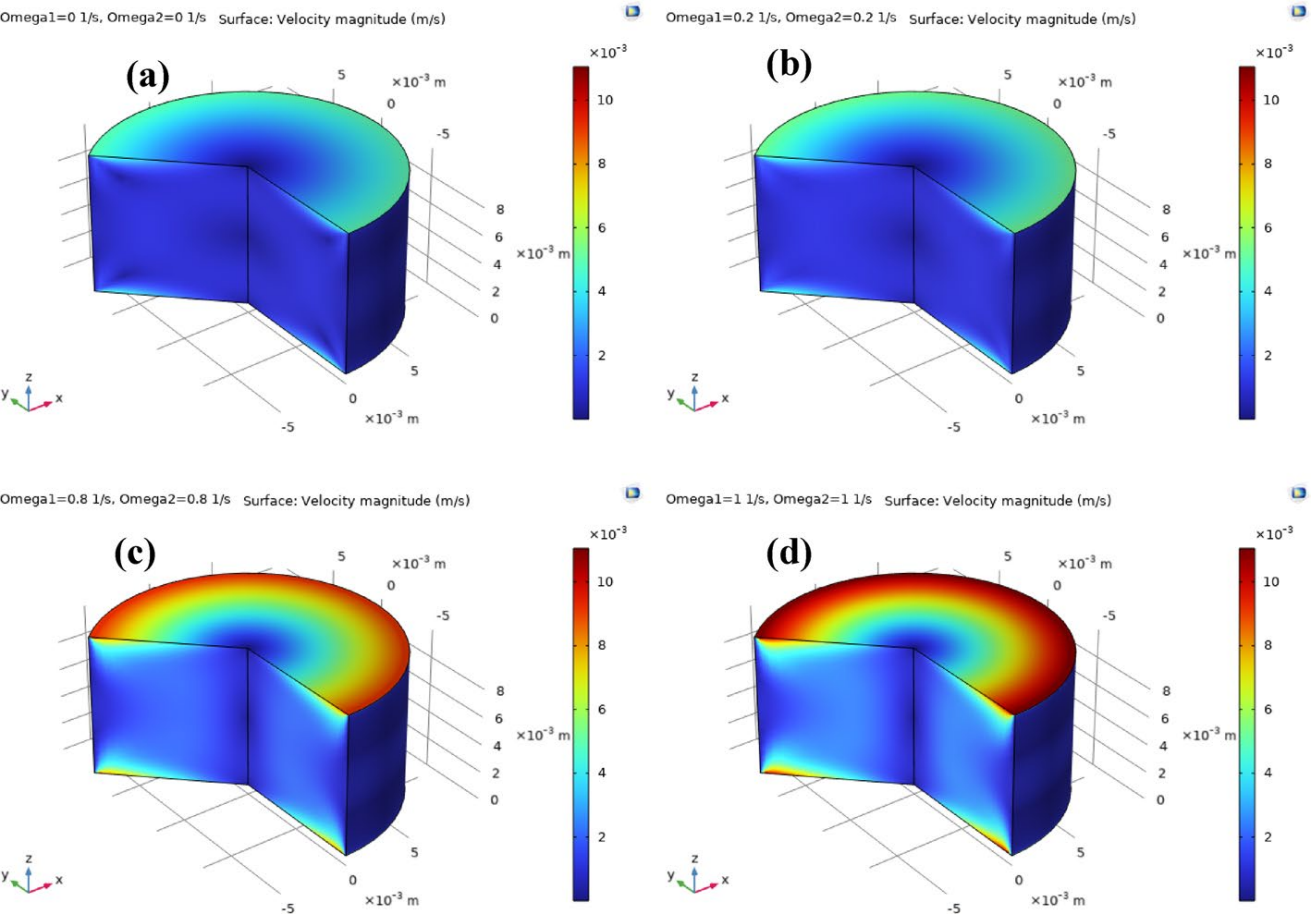
**a**–**d**: velocity magnitude against ${\Omega _1}={\Omega _2}$. Velocity magnitude distribution of ternary nanofluid between coaxial disks for increasing disk rotation rates ${\Omega _1}={\Omega _2}$. **a** ${\Omega _1}={\Omega _2}=0$ (stationary disks), **b** ${\Omega _1}={\Omega _2}=0.2$, **c** ${\Omega _1}={\Omega _2}=0.8$, and **d** ${\Omega _1}={\Omega _2}=1.0$. The results show progressive enhancement of azimuthal motion and higher velocity magnitudes near the disk rims as rotation speed increases

**Fig. 12:**
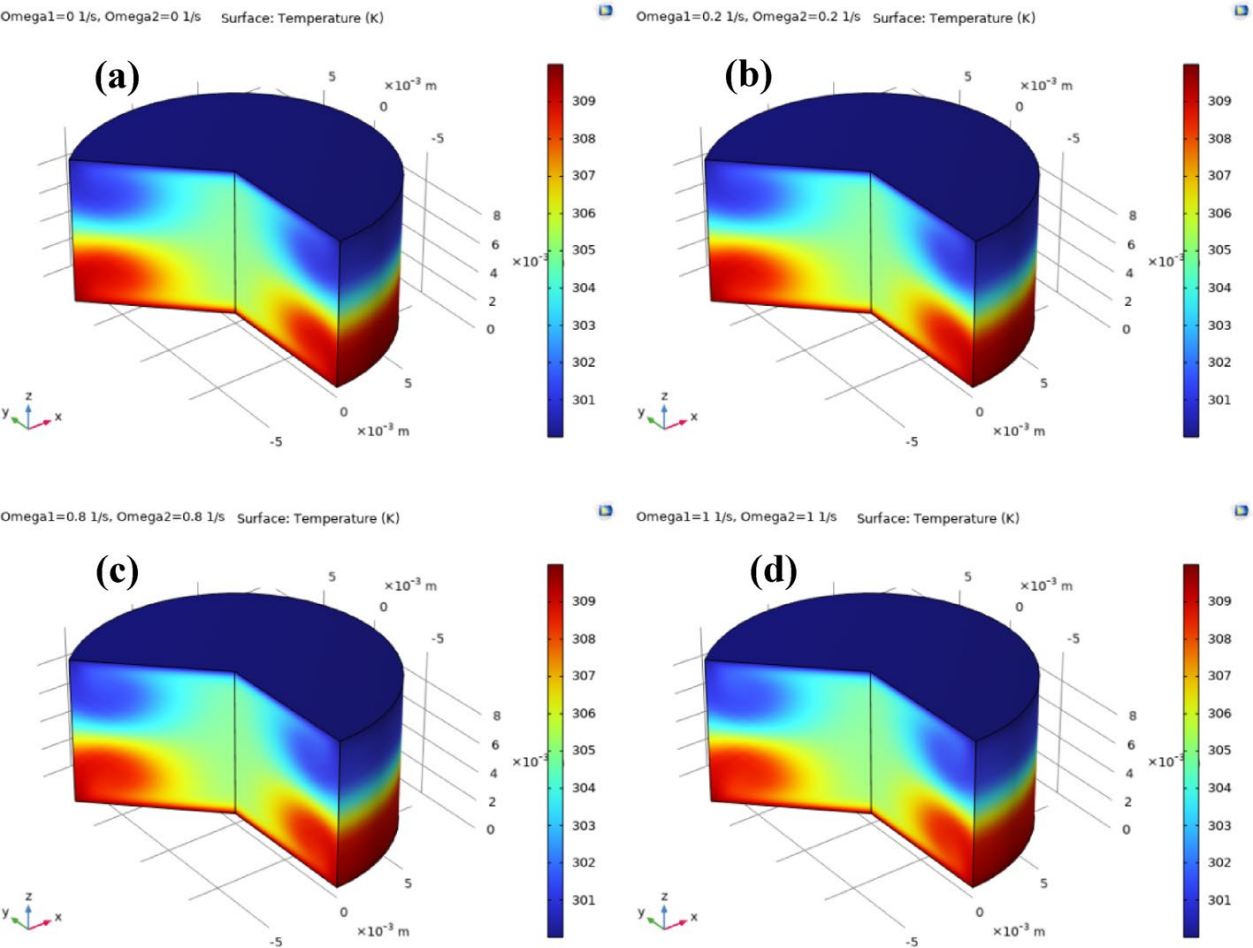
**a**–**d**: temperature distribution against ${\Omega _1}={\Omega _2}$. Temperature distribution of ternary nanofluid between coaxial disks for increasing disk rotation rates ${\Omega _1}={\Omega _2}$. As rotation intensifies, stronger convective motion redistributes heat, leading to thicker thermal boundary layers, enhanced mixing, and more uniform temperature fields

**Fig. 13:**
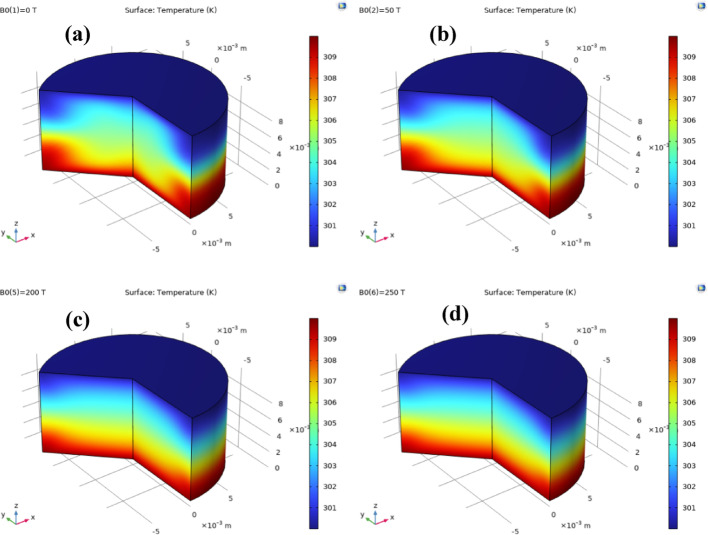
**a**–**d**: velocity magnitude against ${B_0}$. Effect of magnetic field strength ${B_0}$​ on velocity magnitude of ternary nanofluid between rotating disks. **a** ${B_0}=0$, **b** ${B_0}=50$, **c** ${B_0}=200$, and **d** ${B_0}=250$. Increasing magnetic parameter produces a noticeable damping of the flow field, with reduced velocity magnitudes and thinner velocity layers, demonstrating the retarding influence of Lorentz forces on nanofluid motion

**Fig. 14:**
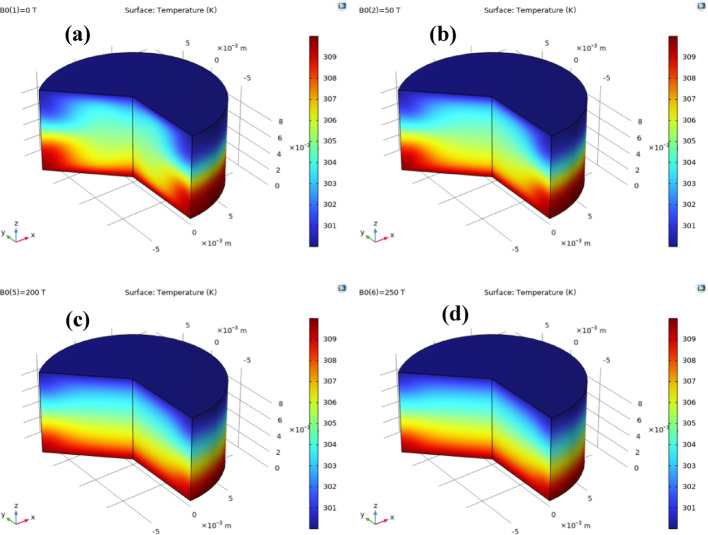
**a**–**d**: temperature distribution against ${B_0}$. Influence of magnetic field strength ${B_0}$ on the temperature distribution of ternary nanofluid between rotating disks. As the magnetic parameter increases, the velocity suppression by Lorentz forces reduces convective transport, resulting in thicker thermal boundary layers and higher fluid temperatures near the heated disk surfaces

**Fig. 15:**
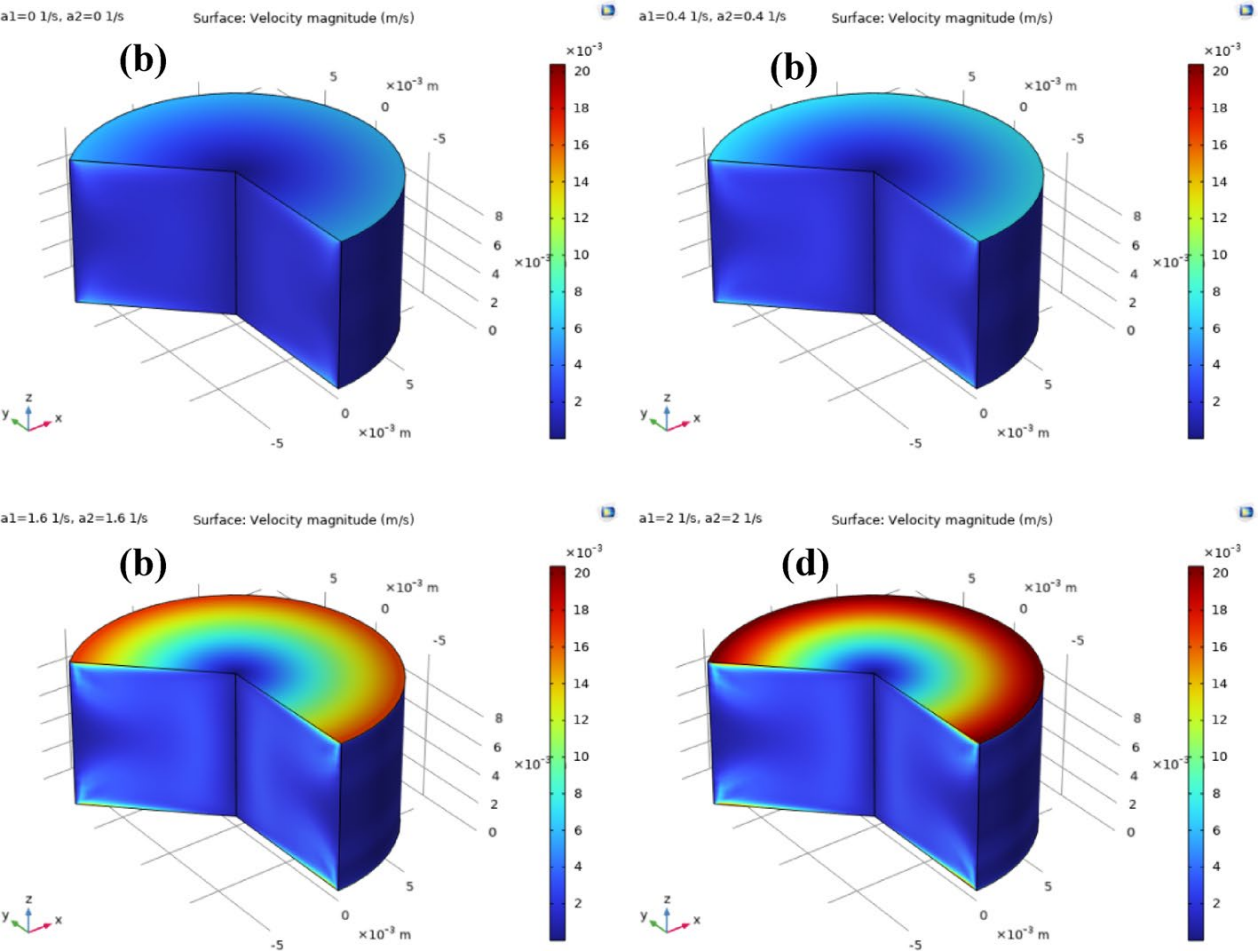
**a**–**d**: velocity magnitude against ${a_1}={a_2}$ . Velocity magnitude distribution of ternary nanofluid between coaxial disks under varying stretching rates of the disk surfaces. **a** ${a_1}={a_2}=0$, **b** ${a_1}={a_2}=0.4$, **c** ${a_1}={a_2}=1.6$, and **d** ${a_1}={a_2}=2$. Increasing the stretching rate intensifies radial outflow and enhances the overall velocity magnitude, with higher values concentrated near the disk rims due to stronger stretching-induced momentum transfer

**Fig. 16:**
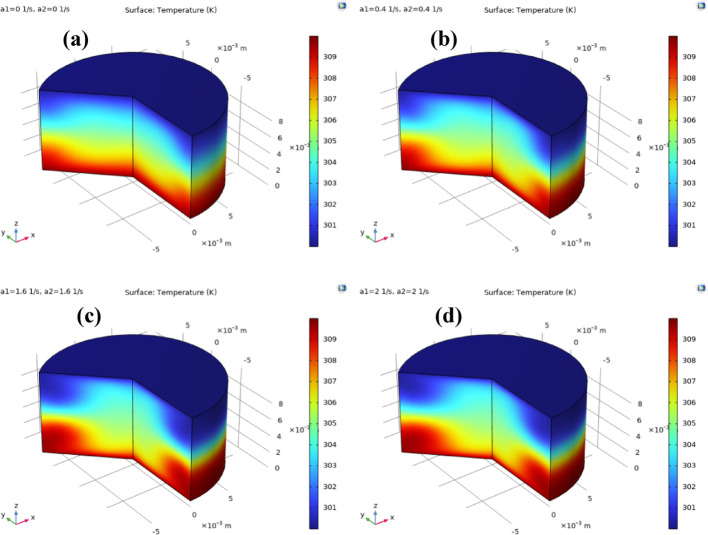
**a**–**d**: temperature distribution against ${a_1}={a_2}$. Temperature distribution of ternary nanofluid between coaxial disks for different stretching rates. Increasing the stretching rates enhances radial fluid motion and convective transport, which redistributes heat more effectively across the gap. As a result, the thermal boundary layers thicken and temperature gradients near the heated disk surfaces are reduced, indicating improved heat transfer performance at higher stretching intensities

Table 4 represents skin friction for Upper and Lower Surfaces against Identical Parameters. The Forchheimer drag parameter (Fr) represents the inertial resistance due to the presence of a porous medium. As Fr increases, skin friction decreases because the increased resistance reduces fluid velocity near the disk surfaces. The ternary nanofluid exhibits the lowest skin friction due to its superior momentum-carrying capacity, followed by the hybrid and simple nanofluids. This behavior highlights the enhanced flow resistance in nanofluids with higher particle concentrations. The magnetic parameter M reflects the opposing force exerted by the magnetic field on the fluid flow, known as the Lorentz force. As M increases, skin friction increases because the magnetic field resists fluid motion. The ternary nanofluid shows the most significant increase in skin friction, which is due to its higher viscosity and enhanced interaction with the magnetic field. This result emphasizes the influence of the magnetic field on flow resistance, especially in highly viscous nanofluids. The rotation ratio Ω∗ affects the shear stress between the two disks. As Ω∗ increases, skin friction increases due to enhanced shear from the rotational motion. This effect is more prominent in ternary nanofluids, which exhibit the highest increase in skin friction. The enhanced rotation speeds lead to more shear between the fluid and the disk surfaces, thus increasing the resistance to flow. Higher slip parameters Lu∗ and Lv∗ reduce the skin friction by allowing the fluid to slip more easily near the boundary, reducing resistance. The ternary nanofluid shows the least decrease in skin friction due to its higher viscosity, which limits the effectiveness of slip in reducing resistance. As the slip parameters increase, the skin friction decreases, but the ternary nanofluid still exhibits higher resistance due to its enhanced interaction between the nanoparticles and the fluid.

**Table 4:** Skin friction for upper and lower surfaces (identical parameters)

Parameters	Effect on skin friction	Tuning	Simple nanofluid	Hybrid nanofluid	Ternary nanofluid
*Fr*	Decrease $\downarrow$	0.6, 0.9, 1.2	0.35, 0.33, 0.30	0.25, 0.23, 0.22	0.15, 0.12, 0.10
*M*	Increase $\uparrow$	1.5, 1.8, 2.1	1.2, 1.3, 1.4	1.5, 1.6, 1.7	2.1, 2.2, 2.3
${\Omega ^*}$	Increase $\uparrow$	1.1, 1.3, 1.5	1.5, 1.6, 1.7	2.0, 2.2, 2.3	2.5, 2.7, 2.8
$L_{u}^{*}$	Decrease $\downarrow$	0, 0.1, 0.2	0.2, 0.18, 0.15	0.1, 0.08, 0.06	0.05, 0.04, 0.03
$L_{v}^{*}$	Decrease $\downarrow$	0, 0.1, 0.2	0.2, 0.18, 0.15	0.1, 0.08, 0.06	0.05, 0.04, 0.03
${\delta _1}$	Increase $\uparrow$	1, 1.2, 1.4	0.38, 0.36, 0.33	0.28, 0.27, 0.25	0.18, 0.17, 0.15
${\delta _2}$	Increase $\uparrow$	1, 1.2, 1.4	0.37, 0.35, 0.32	0.27, 0.26, 0.24	0.17, 0.16, 0.14

Table 5 represents Nusselt number for Upper and Lower Surfaces against Identical Parameters. As the Forchheimer drag increases, the Nusselt number increases for all types of nanofluids. This is because higher drag resistance leads to more viscous dissipation in the system, which enhances heat transfer. The ternary nanofluid consistently exhibits the highest Nusselt numbers across all parameter variations due to its superior heat conduction properties. The magnetic parameter also increases the Nusselt number, as the magnetic field induces higher resistance to flow, leading to more viscous dissipation and enhanced thermal energy generation. Ternary nanofluids once again show the highest values, due to their higher viscosity and nanoparticle content that interact more strongly with the magnetic field. The Eckert number, which represents the conversion of mechanical energy into thermal energy, leads to an increase in the Nusselt number. This occurs because more mechanical energy is converted into heat, which increases the temperature gradient and improves heat transfer. The ternary nanofluid exhibits the highest Nusselt number, highlighting its better thermal conductivity and energy dissipation capabilities. The heat sink parameter reduces the Nusselt number, as higher Q∗ means more heat is absorbed by the system, which reduces the temperature gradient across the fluid. The ternary nanofluid still performs the best in heat transfer efficiency, even under heat absorption conditions, due to its superior thermal properties. An increase in the rotation ratio enhances shear stress and turbulent mixing, which improves heat transfer. The ternary nanofluid benefits the most from this enhancement, maintaining the highest Nusselt numbers in all cases. Higher slip parameters decrease the Nusselt number as slip reduces the resistance near the boundary and limits the thermal boundary layer formation. However, the ternary nanofluid, with its higher viscosity, shows the least decrease in Nusselt number, maintaining more effective heat transfer compared to simple and hybrid nanofluids. Stretching ratios at both the upper and lower disks enhance radial heat transfer by increasing the shear stress near the disk surfaces. The ternary nanofluid shows the highest Nusselt number in these cases due to its better thermal conductivity and ability to transfer heat more efficiently compared to simple and hybrid nanofluids.

**Table 5:** Nusselt number for upper and lower surfaces (identical parameters)

Parameters	Effect on skin friction	Tuning	Simple nanofluid	Hybrid nanofluid	Ternary nanofluid
*Fr*	Decrease $\downarrow$	0.6, 0.9, 1.2	1.8, 1.75, 1.6	2.0, 1.9, 1.85	2.5, 2.3, 2.1
*M*	Increase $\uparrow$	1.5, 1.8, 2.1	2.1, 2.15, 2.2	2.4, 2.45, 2.5	2.8, 2.9, 3.0
*Ec*	Increases $\uparrow$	0.4, 0.6, 0.8	2.0, 2.1, 2.2	2.2, 2.3, 2.4	2.7, 2.8, 2.9
${Q^*}$	Decrease $\downarrow$	0.5, 0.7, 0.9	1.8, 1.75, 1.7	1.7, 1.6, 1.5	1.5, 1.4, 1.3
${\Omega ^*}$	Increase $\uparrow$	1.1, 1.3, 1.5	1.6, 1.7, 1.8	1.9, 2.0, 2.1	2.3, 2.4, 2.5
$L_{T}^{*}$	Decrease $\downarrow$	0, 0.1, 0.2	1.5, 1.45, 1.4	1.6, 1.55, 1.5	1.7, 1.65, 1.6
${\delta _1}$	Increase $\uparrow$	1, 1.2, 1.4	2.0, 2.05, 2.1	2.2, 2.3, 2.4	2.6, 2.7, 2.8
${\delta _2}$	Increase $\uparrow$	1, 1.2, 1.4	1.95, 2.0, 2.05	2.1, 2.2, 2.3	2.5, 2.6, 2.7

## Conclusion

In this study, we presented a comprehensive analysis of the flow and heat transfer characteristics of simple, hybrid, and ternary nanofluids between rotating and stretching disks embedded in a porous medium. This model considered the influence of magnetic fields, viscous dissipation, and Cattaneo–Christov thermal relaxation. The model incorporated slip conditions, disk stretching ratios, and Forchheimer drag to enable a realistic simulation of practical engineering systems that utilize a enhanced heat transfer fluid.

The key findings can be summarized as follows:

1. Increasing Fr suppressed both axial and radial velocities due to inertial resistance in the porous medium, while enhancing thermal energy accumulation, leading to higher temperatures. Among the fluids, ternary nanofluids show superior momentum retention despite increased resistance.
2. The Lorentz force reduced flow velocities in all directions, with ternary nanofluids experiencing the highest suppression. Simultaneously, the temperature increased due to damped convection to indicate the dual role of MHD in flow control and heat retention.
3. Higher rotation ratios intensify shear stresses and enhanced velocities, particularly in radial and azimuthal directions, to improve the Nusselt number through increased convective transport. The ternary nanofluid benefits the most due to its high viscosity and nanoparticle-induced momentum.
4. Radial stretching significantly influenced the radial velocity and radial skin friction, while axial, azimuthal velocities, and temperature profiles remain largely unaffected. Higher stretching ratios enhanced the radial momentum and heat transfer, with the ternary nanofluid consistently outperforming hybrid and simple nanofluids.
5. Slip reduced the velocity gradient and skin friction near the boundaries, slightly lowering the heat transfer rate. Nevertheless, ternary nanofluids maintain superior Nusselt numbers due to their higher thermal conductivity, illustrating their robustness in practical applications.
6. The Nusselt number consistently increased with Eckert number, rotation ratio, and stretching ratio, while it decreased with higher heat sink parameter Q∗. The ternary nanofluid exhibits the highest Nusselt numbers, demonstrating its superior capacity for heat transport compared to hybrid and simple nanofluids.
7. The ternary nanofluid experienced higher wall shear stresses due to its enhanced viscosity and momentum transport properties, particularly under higher magnetic field strength, Eckert number, and rotation ratios, suggesting stronger flow resistance in practical systems.

Overall, this study highlights the enhanced momentum and thermal performance of ternary nanofluids under combined MHD, porous, and stretching disk effects, offering significant improvements over hybrid and simple nanofluids. These findings provide valuable insights for designing advanced cooling and heat transfer systems, micro- and nano-scale devices, and industrial applications where enhanced heat transfer is critical.

The results also demonstrated the sensitivity of flow and heat transfer to key parameters such as magnetic field strength, disk stretching, and slip, providing a versatile framework for optimizing nanofluid-based thermal management systems. Future work could extend this model to non-spherical nanoparticles, transient flow conditions, and radiative heat transfer, opening avenues for more efficient thermal and energy management in engineering applications.

## Data Availability

The datasets generated and analyzed during the current study are available from the corresponding author on reasonable request.
